# A systems biology approach to dynamic modeling and inter-subject variability of statin pharmacokinetics in human hepatocytes

**DOI:** 10.1186/1752-0509-5-66

**Published:** 2011-05-06

**Authors:** Joachim Bucher, Stephan Riedmaier, Anke Schnabel, Katrin Marcus, Gabriele Vacun, Thomas S Weiss, Wolfgang E Thasler, Andreas K Nüssler, Ulrich M Zanger, Matthias Reuss

**Affiliations:** 1 Institute of Biochemical Engineering, Allmandring 31, and Center Systems Biology, Nobelstraße 15, University of Stuttgart, 70569 Stuttgart, Germany; 2 Dr. Margarete Fischer-Bosch Institute of Clinical Pharmacology, Auerbachstraße 112, 70376 Stuttgart, and University of Tübingen, 72074 Tübingen, Germany; 3 Dep. Functional Proteomics, Medizinisches Proteom-Center, Ruhr-University Bochum, Universitätsstraße 150, 44801 Bochum, Germany; 4 Department of Surgery, University of Regensburg, F.J.S. Allee 11, 93053 Regensburg, Germany; 5 Department of Surgery, Ludwig-Maximilians-University, Marchioninistraße 15, 81377 München, Germany; 6 Department of Traumatology, Technical University of Munich, MRI, Ismaningerstraße 22, 81675 Munich, Germany; 7 Insilico Biotechnology AG, Meitnerstraße 8, 70563 Stuttgart, Germany; 8 Fraunhofer Institut für Grenzflächen und Bioverfahrenstechnik, 70569 Stuttgart, Germany

## Abstract

**Background:**

The individual character of pharmacokinetics is of great importance in the risk assessment of new drug leads in pharmacological research. Amongst others, it is severely influenced by the properties and inter-individual variability of the enzymes and transporters of the drug detoxification system of the liver. Predicting individual drug biotransformation capacity requires quantitative and detailed models.

**Results:**

In this contribution we present the *de novo *deterministic modeling of atorvastatin biotransformation based on comprehensive published knowledge on involved metabolic and transport pathways as well as physicochemical properties. The model was evaluated on primary human hepatocytes and parameter identifiability analysis was performed under multiple experimental constraints. Dynamic simulations of atorvastatin biotransformation considering the inter-individual variability of the two major involved enzymes CYP3A4 and UGT1A3 based on quantitative protein expression data in a large human liver bank (n = 150) highlighted the variability in the individual biotransformation profiles and therefore also points to the individuality of pharmacokinetics.

**Conclusions:**

A dynamic model for the biotransformation of atorvastatin has been developed using quantitative metabolite measurements in primary human hepatocytes. The model comprises kinetics for transport processes and metabolic enzymes as well as population liver expression data allowing us to assess the impact of inter-individual variability of concentrations of key proteins. Application of computational tools for parameter sensitivity analysis enabled us to considerably improve the validity of the model and to create a consistent framework for precise computer-aided simulations in toxicology.

## Background

The discovery and development of new drug entities is strongly handicapped by the circumstance that about 50% of the drug candidates fail in the clinical studies [1]. About one quarter of candidate drugs fail due to toxicity or pharmacokinetic (PK) problems [2], and currently, it is a well known fact, that toxicity is the major cause of attrition in the drug development process [3]. Therefore, it is quite clear that dangerous properties of drug entities have to be revealed very early in the drug evaluation studies [4]. Despite the ever growing effort to apply computational power towards improving the understanding and *in-silico *prediction of drug pharmacokinetics and response, the overall impact on preclinical safety testing has been modest.

Application of systems biology holds tremendous promise because it aims to understand and quantitatively describe biological phenomena within the framework of the hierarchical levels of metabolic pathways and regulatory networks at the different scales of cells, tissue, organs and ultimately whole organisms [5,6]. However, despite emerging consensus that such a holistic approach is essential to provide the framework of predictive toxicology, the number of successful case studies is still minuscule [7-9].

Current activities can be grouped into

(1) quantitative structure-activity-relationship (QSAR) methods, computational models based on compound structure and focused on potential interactions of small molecules with major classes of proteins such as drug metabolizing enzymes [10-15], transporters [16] and receptors [16-18]. Also important are physicochemical properties of the drug, for example solubility and permeability that are estimated from the molecular structure [19-22].

(2) in vitro kinetics for prediction of in vivo drug clearance using kinetic data from recombinant cytochromes P450 (CYPs), microsomes and hepatocytes (IVIVE: in vitro-in vivo extrapolations) [23].

(3) physiologically based PK (PBPK) modeling [24-28] which considers the anatomical, physiological and chemical aspects of ADME (absorption, distribution, metabolism and elimination of the drug) [29-31] in multi-compartment models [32].

In addition to these simulations based on mathematical models various computational and bioinformatics approaches are applied to extract information from high throughput data of drug response experiments at cellular, tissue, organ and whole organism level.

A critical assessment of the aforementioned tools, essentially to outline gaps that must be addressed for more reliable predictive simulation-based toxicology, indicates needs for more rigorous network models focusing at systems dynamics beyond kinetics of individual enzymes, consideration of inter-individual variability and systematic investigations of parameter sensitivity and its impact on model verification, discrimination and reduction, to name a few. The first issue is related to the design of the dynamic models for the drug elimination process in the hepatocyte, which should be based on the integration of membrane transport processes for substrates and products as well as phase I and phase II reactions. These models need to be tightly linked to stimulus (dose)-response experiments.

The issue of model parameterization in the context of modeling in toxicology has been already addressed in 1995 by Andersen et al [24]. In addition to the problem of identifiability, particular attention should be given to correlation between parameters, very common in biological systems.

Yet another question of interest concerns the subtle integration of the enormous inter-subject variability in enzymatic phenotypes into the model. This is of outermost importance for predictions in toxicology and also in clinical pharmacology in order to design optimal treatments for individual patients. Consideration of this variability in phenotype should rest on quantitative proteomics and activity data from human liver tissue or hepatocytes representing a statistically significant portion of the population.

The importance of this issue has been extensively addressed in the review of Rostami-Hodjegan and Tucker [23] and discussed in the context of IVIVE (in vitro-in vivo extrapolation) approach. Significant progress has been made by this group through a strategy which is based on the simulation of drug disposition in virtual liver populations. The basic idea of this method is to portray the variability into the *r*_*max*_/*K*_*M*_-values estimated from in vitro studies using human liver microsomes, primary or cryopreserved hepatocytes or recombinant expressed enzymes. This is a promising approach; however, improvement of the strategy is imaginable by separation of the two parameters *r*_*max *_and *K*_*M *_(maximal enzymatic rate and enzymatic affinity parameter in Michaelis-Menten- and derived kinetics).

This concept, which is a focus of the present article, is driven by the possibility to incorporate separate information from pharmacokinetics and quantitative proteomics as well as future incorporation of the regulatory network responsible for variation of expression level of the enzymes, transporters and receptors. As model drug we chose atorvastatin (AS), one of the most frequently used 3-hydroxymethylglutaryl coenzyme A reductase inhibitors (statins). Statins thereby reduce cholesterol synthesis and they also stimulate the uptake of LDL-cholesterol from the blood. Although they are relatively safe drugs, the lipid-lowering effect of statins is inadequate in some patients, and unpredictable drug-drug interactions can occur, as well as hepatic and extrahepatic adverse effects including hepatotoxicity, myopathy, and rare but severe rhabdomyolysis.

Some aspects of dynamic modeling of statins have been tackled in previous studies, including the deterministic modeling of transport kinetics [33,34] and the pharmacokinetic modeling of the clearance mechanisms including consideration of unspecific binding effects [35]. In the latter case, a multi-compartmental model approach has been derived on isolated rat hepatocytes. However, the metabolism of AS inside the liver cell has not been described in details, missing the kinetic description of the different metabolite formations, which thus far precludes the consideration of inter-individual variability, apart from the fact that rat hepatocytes had been investigated.

This study describes a deterministic modeling approach of the dynamic biotransformation and transport processes of AS in human hepatocytes considering both kinetics of transport processes as well as intracellular detoxification processes via Phase I and Phase II enzymes. Attention is given to the detailed enzymatic and chemical reactions, including conversions between the acid and lactone form of AS as well as unspecific binding between metabolites and macromolecules like proteins. The additional integration of quantitative protein data of Phase I and Phase II enzymes enables the dynamic analysis of the underlying inter-individual-variability of expression levels of these enzymes.

## Methods

### Chemicals

Williams Medium E (WME) without phenol-red and without L-glutamine and stabilized L-glutamine were obtained from Pan-Biotech-GmbH (Aidenbach, Germany). Penicillin/Streptomycin and ITS-X were obtained from Invitrogen (Karlsruhe, Germany). Bovine serum albumin (BSA) and dimethyl sulfoxide (DMSO) were purchased from Sigma-Aldrich Chemicals (Taufkirchen, Germany). Dexa Inject was obtained from Jenapharm (Jena, Germany). AS, its metabolites and deuterated standards were purchased from Toronto Research Chemicals Inc. (North York, Canada). PBS was obtained from Invitrogen (Karlsruhe, Germany) and Complete Mini, EDTA-free from Roche Diagnostics (Mannheim, Germany). NaPP, sucrose and LiChrosolv were purchased from Merck (Darmstadt, Germany); acetonitrile from Roth (Karlsruhe, Germany), formic acid from Fluka, (Germany), UGT1A3 monoclonal mouse antibody from Abcam (Cambridge, England). Trypsin was purchased from Promega (Mannheim, Germany). Synthetic peptides were purchased from Sigma-Genosys (Haverhill, UK).

### Isolation and cultivation of primary human hepatocytes

Tissue samples from human liver resections were obtained from patients undergoing partial hepatectomy. Experimental procedures were performed according to the guidelines of the charitable state-controlled foundation HTCR (Human Tissue and Cell Research) Regensburg, Germany, and the institutional guidelines for liver resections of tumor patients with primary or secondary liver tumors, Technical University Munich, MRI, Munich, Germany. The use of human hepatocytes for research purposes was approved by the local ethics committees of the Ludwig-Maximilians-University of Munich [36] and the Charité, Humboldt University Berlin [37], Germany, and written informed consent was obtained from all patients. Hepatocytes were cultured on collagen gel precoated 6-well plates at a density of 1.5·10^6 ^cells/well. Cells were allowed to attach to the collagen layer. After transport, culture media was disposed and attached cells were cultured 24 h at 37°C in a humidified chamber with 95%/5% air/CO2 in serum-free medium WME, supplemented with albumin (0.1% (v/v)), penicillin/streptomycin (100 U/ml), stabilized L-glutamine (2 mM), dexamethasone dihydrogenphosphate (0.025% (v/v)) and ITS-X (5 mg insulin, 3.35 μg natrium-selenit, 2.75 mg transferrin and 1 mg ethanolamine), further named SFM.

### Time-series experiments

Incubation with AS was started by disposing the culture media and cultivation of the attached cells at 37°C in a humidified chamber with 95%/5% air/CO_2 _in 2 ml SFM, supplemented with 10 μM AS, 0.1% (v/v) BSA and 0.1% DMSO. At specified time-points, three wells were further treated for the preparation of samples for the measurement of extracellular and intracellular metabolites, respectively. SFM media was collected and 50 μL formic acid and deuterated internal standard was added for the further measurement of extracellular metabolites. Cells were harvested in pre-cooled albumin-free SFM, disrupted by freeze/thaw and ultra-sonification and centrifuged. The supernatant was used for the determination of intracellular metabolites.

For the preparation of samples for the protein measurements, culture medium was disposed and cells were harvested in pre-cooled PBS, supplemented with Complete Mini EDTA-free (1 Tablet/10 ml Buffer). Cell suspensions were centrifuged 5 min (500 g) at 4°C and cell pellets were resuspended in 150 μL NaPP-buffer (0.1 M, pH 7.4), containing 250 mM sucrose and Complete Mini EDTA-free (1 Tablet/10 ml Buffer). Cells were disrupted by ultra-sonification and lyophilized for the analysis of total protein concentration, CYP3A4 and UGT1A3 content.

For cell number determination, cells from two wells were fixed with methanol-acetic acid fixative solution (10 min at 37°C and 4°C) and afterwards nuclei were stained for 15 min with Meyers Hämalaun (Sigma-Aldrich Chemie GmbH, Germany), rinsed with water and air-dried. Stained nuclei were counted in digital images (10 per well) at 40-fold Magnification (ImageJ Image Processing and Analysis Program).

### Quantification of atorvastatin and its metabolites

AS and ASL, and their para- (ASpOH, ASLpOH) and ortho-hydroxy-metabolites (ASoOH, ASLoOH), were determined by LC-MS-MS analysis using the respective deuterium labeled analogues as internal standards, essentially as described [38]. HPLC separation was performed at 30°C on a XBridge Shield RP18 column (2.1 × 50 mm, 3.5 μm, Waters) using (A) 1 mM formic acid and (B) acetonitrile as mobile phases at a flow rate of 0.4 ml/min. Gradients were programmed as follows: 63% A for 4 min; linear decrease of A to 60% within 9 min; linear decrease of A to 55% within 2.5 min; 55% A for 1 min; increase of A to 63% in 0.2 min. Equilibration time of the column was 20 min. MS-MS analysis was performed on an Esquire HCT ultra ion trap mass spectrometer (Bruker Daltonics, Bremen, Germany) coupled to an HPLC 1100-System (Agilent, Waldbronn, Germany) consisting of binary pump G1312A, degasser G1379A, well-plate sampler G1367A and column thermostat G1330B. The ionization mode was electrospray (ESI), polarity positive, mass range mode ultrascan, and nitrogen was used as a drying and nebulizer gas. The following parameters were applied: nebulizer 45 psi, dry gas 10 l/min, dry temperature 300°C, capillary 4100 V, scan range 200 - 600 *m/z*.

Precursor and product ions (m/z) of analytes and internal standards, respectively, were ATV (559 and 440.2; 466.2), [^2^H_5_]ATV (564 and 445.2; 471.2), ATV-L (541.2 and 448.2), [^2^H_5_]ATV-L (546.2 and 453.2), p-OH-ATV (575 and 440.2; 466.2), [^2^H_5_]p-OH-ATV (580 and 445.2; 471.2), p-OH-ATV-L (557 and 448.4), [^2^H_5_]p-OH-ATV-L (562 and 448.4), o-OH-ATV (575 and 466.4), [^2^H_5_]o-OH-ATV (580 and 471.2), o-OH-ATV-L (557 and 448.4), [^2^H_5_]o-OH-ATV-L (562 and 448.4). Sample quantification was possible in a range from 0.5 to 500 pmol.

### CYP3A4 and UGT1A3 protein quantification

Protein quantification of CYP3A4 and UGT1A3 in human liver microsomes and relative protein quantification of UGT1A3 in lyophilized samples of primary hepatocytes was performed by immunoblotting as described previously [38,39].

Cell lysates for absolute quantification analysis of CYP3A4 were prepared from lyophilized human primary hepatocytes by sonification in the presence of glass beads in buffer containing Complete Mini-Protease Inhibitor Cocktail, and following homogenization. In the CYP3A4 quantification assay, three synthetic isotopically labeled peptides (13C/15N amino acid) were used as internal standard for calibration. These isotope labeled standard peptides represented sequence analogues to proteotypic peptides of CYP3A4, which arise from tryptic digestion. After acetone precipitation and resolving of the proteins in 8 M Urea, a definite amount of internal standard peptides was added. The sample mixture was reduced with 5 mM DTT and alkylated with 15 mM iodacetamide in 50 mM ammonium bicarbonate. Subsequently samples were digested with trypsin at 42°C for 4 h (enzyme/substrate ratio of 1:10). Efficiency of tryptic digestion was checked by SDS-PAGE followed by silver staining. The resulting peptides were purified using C18 OMIX^® ^Tips (Varian, Darmstadt, Germany) according to manufacturer's suggested protocol and separated on a nanoliter-flow Ultimate HPLC system (Dionex, Idstein, Germany). After injection (15 μl), peptides were trapped and desalted on a precolumn (0.3 mm I.D. × 5 mm PepMapTM, Dionex) at a flow rate of 30 μl/min in 0.1% TFA for 6 min. Peptides were transferred to the separation column (75 μm I.D. × 250 mm PepMapTM column, Dionex) and separated in a linear gradient of mobile phase (A: 0.1% formic acid, B: 84% acetonitrile/0.1% formic acid) from 5% B to 35% B over a period of 35 min with a flow of 290 nl/min. The column effluent was continuously directed into the NanoSpray II source of a 4000QTrap mass spectrometer (Applied Biosystems, Foster City, CA, USA). The MS was set up to run a multiple reaction monitoring experiment essentially, as described previously [40], including two to three parent-to-product ion transitions for each internal standard peptide as well as the corresponding transitions of native peptide of CYP3A4. The instrument settings were as follows: ion spray voltage, 3-4 kV; interface heater, 150°C; declustering potential, 50 V; collision energy, peptide specific; entrance potential, 10 V; collision cell exit potential, 10 V. MS data were processed by integrating the appropriate peak areas from extracted ion chromatograms by MultiQuantTM Software (Applied Biosystems). The absolute amount of CYP3A4 protein was calculated from the peak area ratio "internal standard peptide/native peptide". Total protein content of the samples were determined by amino acid analysis (AAA) on a Waters 2695 HPLC system using the AccQ•Tag derivatization method (Waters, Eschborn, Germany), according to manufacturer's instructions.

### Identification of metabolic network structure

Numerous metabolic and physicochemical aspects about AS had to be considered in the initial model building. AS exists in two forms, a very lipophilic lactone (ASL) and a comparably hydrophilic hydroxyl-acid (AS). AS is converted enzymatically via an instable intermediate product into ASL, mediated by different UGT isoenzymes [41,42]. Recent investigations by ourselves and others have shown that the most important contributor to UGT-driven lactonization is UGT1A3, whereas UGT1A1 plays an insignificant role [38,42]. AS acids and lactones are inter-converted chemically into each other [43]. However, studies have indicated, that the chemical lactonization of AS to ASL can be neglected at physiological pH of 7.4 [43]. Recent studies highlight, that different PON enzymes might also be possible contributors to the lactone hydrolysis and that PON1 is present in liver [44-48].

Both AS and ASL are hydroxylated in human hepatocytes leading to para- and ortho-hydroxy-metabolites, ASpOH, ASoOH, ASLpOH and ASLoOH [49,50], mainly catalyzed by CYP3A4 [51]. Recent studies have reported, that CYP2C8 and CYP3A5 also hydroxylate AS to a minor extent [50,52].

Furthermore, AS is transported into the cell via organic anion transport polypeptides (OATP), and recent studies on recombinant systems showed, that OATP1B1 and OATP2B1 contribute to the AS import [53,54], which are both expressed in the human liver [55,56]

OATP1B3 is supposed to be also a main contributor to drug transport [54], since it shows high gene expression levels in liver [57], but its importance for AS has not been investigated kinetically so far.

OATP transporters have been reported to be bidirectional facilitated diffusion transporters, independent from ATP and Na+, K+ and H+, but with a possible involvement of reduced glutathione [58,59]. However, previous investigations on transport mechanisms on rat hepatocytes, showed, that the intracellular concentrations of pitavastatin and other compounds are much higher than outside the cell [33,34,60]. Facilitated or passive diffusion do not allow a greater intracellular than extracellular concentration of the parent drug of interest, when the source is the initial extracellular concentration, because it is a concentration-gradient dependent mechanism. Therefore, the import mechanism should be rather considered as active mediated transport, which is not concentration-gradient dependent, rather than as the proposed facilitated diffusion process.

As shown with the recombinantly expressed transporters mentioned above [54], transport of the acidic metabolites, ASpOH and ASoOH, by OATP1B1 was similar to that of AS, and transport of the corresponding lactones was also mediated by this transporter, although at somewhat lower rates. We therefore assumed that the same OATP transporters are responsible for the import of AS and its hydroxylated and lactone metabolites into hepatocytes.

AS and its hydroxylated metabolites, ASpOH and ASoOH, are actively exported out of the hepatocytes into the bile by the ATP-dependent MDR1 transporter [61,62]. In addition, acidic and lactone form of AS showed inhibitory effects in transport studies of substrates of MDR1 and MRP2 [63-65], pointing to the competitive transport mechanism of these substrates at this proteins. Further, the transporters MRP1, MRP3 and MRP6 are also reported to be responsible for the transport of organic compounds including AS from inside the hepatocytes into the plasma [56,66].

Passive diffusion might play also an important role in the transfer of AS, ASL and the corresponding metabolites, ASpOH and ASoOH, ASLpOH and ASLoOH, respectively. Since the acidic forms of AS are rather hydrophilic and the lactone forms of AS are rather lipophilic, it can be assumed that passive diffusion plays a more important role for the lactone forms, and the transporter mediated active transport plays a more important role for the acidic forms, as reported earlier for statins [67].

Finally, lipophilic drugs have a high affinity to bind non-specifically to proteins, and previous studies concentrated on the modeling of drug binding in the intracellular and the extracellular space as well as on the surface of the cells [60,68,69].

### Mathematical modeling

The mathematical model can be described by the system of non-linear ordinary differential equations(1)

where the change in extracellular, intracellular or unspecific bound metabolite concentration *c*_*j *_in the extra - or intracellular compartment with *V*_*comp *_is effected by the conversion or production of contributing chemical or enzymatic reactions or by transport steps *r*_*ij*_, respectively. The extracellular volume, , equals to the volume of the media used. The intracellular volume, , equals to the total volume of all cells used, and is determined by multiplying the cell number by the volume of a single hepatocyte, estimated to be 14.1 pL by the approximation of a spherical shape with a diameter of 30 μm [70].

Appropriate reaction kinetics *r*_*ij *_are modeled for the CYP3A4 hydroxylation, the UGT1A3 lactonization, the chemical and enzymatic lactone hydrolysis and intracellular unspecific binding to macromolecules.

Previous studies determined substrate inhibition kinetics of the CYP3A4 mediated hydroxylation of AS on human microsomes [52]. However, inhibition effects contribute severely only at a concentration higher than 100 μM. Furthermore, our model approach considers the competitive nature of alternative substrates, by integrating the CYP3A4 hydroxylation of AS and ASL as reaction kinetics describing the competitive conversion of alternative substrates to alternative products, illustrated for the hydroxylation of AS to ASpOH(2)

(see Additional file 1 - Derivation of Atorvastatin kinetics at CYP3A4).

The lactonization of AS to ASL is mediated by UGT1A3 enzymes and the reaction is formulated as substrate inhibition kinetics [41].(3)

The lactone metabolites are either hydrolyzed chemically to the respective acid metabolites [43] inside (*c*) or outside (*m*) the cell, or enzymatically by the contribution of PON enzymes inside the cell. Both reactions are described as first order kinetics(4)

Unspecific binding of intracellular metabolites to macromolecules is formulated as(5)

with the dissociation coefficient *k*_*dis *_and the intracellular fraction unbound [71](6)

which describes the ratio between the intracellular free concentration  to the sum of intracellular free and bound concentration,  and , in equilibrium (index *eq*) (see Additional file 2 - Derivation of kinetics of unspecific protein binding). However, the intracellular free and bound concentrations in equilibrium are not measurable; therefore, the fraction unbound *fu*_*j *_is set as a parameter to be estimated in the parameter optimization procedure.

Transport steps include active import and export of the metabolites as well as passive diffusion steps. Both active import by OATP1B1 or OATP2B1 and export of AS are described as Michaelis-Menten-kinetics [53,54](7)

whereas the active transport kinetics of the other metabolites are assumed to be of first-order [72].(8)

Besides the active transport, metabolites undergo passive diffusion through the double-layer lipid-membrane. Passive diffusion, described as(9)

is driven by the concentration difference between outside and inside the cell, , over the lipid-membrane with thickness *d*, through all cells with the total surface area *A*_*cells*_, and controlled by the diffusion coefficient *D*_*j *_and is comprised as the permeability coefficient *P*_*j*_.

### Optimization procedure for estimation of model parameters

The optimization procedure is based on evolutionary strategies which are implemented with JavaEva (WSI Computer Science Department, Center for Bioinformatics, University of Tübingen, Germany) and a MVA (main vector adaptation) mutation operator [73]. The optimization procedure estimates the parameters in equations (2) to (9) based on the optimization criterion(10)

where the deviation of calculated and measured concentrations divided by the measurement standard deviation *s*_*j*_, squared and summed over all metabolites J and all time points N, has to reach a minimum. Additional optimization constraints are *fu*_*AS *_> *fu*_*ASL*_, *P*_*ASL *_> *P*_*AS*_, *P*_*AS *_> *P*_*ASOH *_and *P*_*ASL *_> *P*_*ASLOH*_, because the lactones have a higher lipophilicity than the acids and the metabolites are supposed to be more hydrophilic than the corresponding parent lactone or acid drug.

The integration of the differential equations (1) using the reaction kinetics in equations (2) to (9) was performed by the differential algebraic equation solver LIMEX (Konrad-Zuse-Zentrum für Informationstechnik, Berlin) [74].

### Relative abundance approach for prediction of *r*_*max*_-parameters

For pharmacokinetic predictions taking inter-individual variability of CYP3A4 and UGT1A3 expression levels into account, maximal rate parameters are predicted via a relative abundance approach, which is based on the assumption that the maximal rate of the reaction is proportional to the enzyme concentration:(11)

The maximal velocities  of the respective enzyme *e*, here CYP3A4 or UGT1A3, in the conversion to the product *P*_*j *_in the liver of interest *li *is estimated from the respective maximal rate  and the enzyme concentration  in the reference liver and the enzyme concentration  in the liver of interest *li*.

### Computational approach

The mathematical model of AS metabolism was coded in FORTRAN language and linked to the numerical integrator LIMEX, also written in FORTRAN. After compilation to the executable program, optimization was started by the call of JavaEva. The mathematical model is supplemented as SBML-file for review purpose.

## Results

### Model-setup

**Figure 1:**
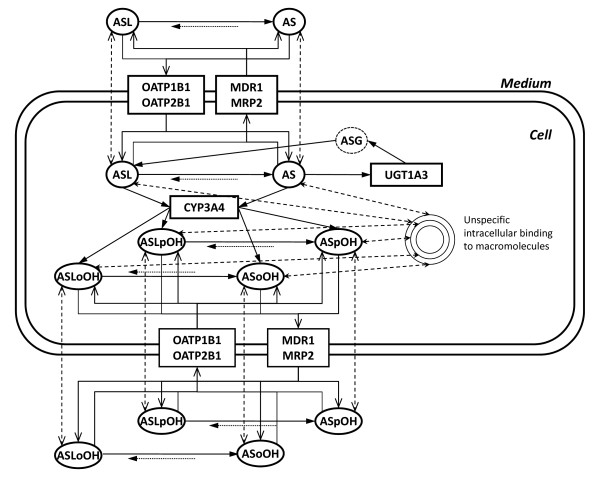
**Scheme of the full model of Atorvastatin metabolism in primary human hepatocytes**. The scheme illustrates atorvastatin metabolic and transport pathways in human hepatocytes. The model comprises the active import and export of AS and ASL, and corresponding para- and ortho-hydroxy-metabolites, ASpOH and ASoOH, ASLpOH and ASLoOH, mediated by import proteins, OATP1B1 and OATP2B1 (solid lines), and export proteins, MDR1 and MRP2 (solid lines), as well as passive diffusion steps (dashed lines). AS and ASL are hydroxylated to the corresponding metabolites by CYP3A4, respectively (solid lines). Compound AS is converted via an instable glucuronid-intermediate to ASL mediated by UGT1A3 (solid lines). The lactones are hydrolyzed either chemically or enzymatically by putative PON to the respective acids (solid and dotted line). Acids and lactones are further subject to unspecific binding to macromolecules in human hepatocytes (dashed lines).

The model structure of AS biotransformation in primary human hepatocytes is schematized in Figure 1 and comprises the description of the extracellular, intracellular and unspecifically bound metabolites, as well as corresponding reaction and transport steps and unspecific protein binding.

Since the calibration and quality samples were prepared in the same media as the experimental metabolite samples and because it can be assumed that the intracellular protein concentration is much higher compared to the outside of the cells, the unspecific protein binding was only considered for the intracellular space.Regarding the unspecific protein binding, it can be assumed that the intracellular protein concentration is much higher compared to the outside of the cells. This assumption is based on the estimation of the ratio of intracellular protein to extracellular media protein concentration. From the total cell protein measurement and the determination of total cell volume a total intracellular protein concentration of about 30 g/l can be estimated. The media includes as sole protein component 0.1% (v/v) albumin, corresponding 1 g/l. Therefore, this assumption is justified.

Because our hepatocyte culture model does not allow to distinguish experimentally import and export, single irreversible, actively mediated transport steps for both directions, as well as passive diffusion steps were considered for each compound, respectively, similar to mechanistic transport models of other studies [33,34]. Only in the case of AS different transport steps for OATP1B1 and OATP2B1 were implemented in the model, because specific KMs had been determined in previous studies [53,54].

### Model verification

Model verification was performed by quantitative measurements of AS and all metabolites in the extra- and intracellular space during time-series experiments, performed first on a single batch of primary hepatocytes from a single individual 1. Special emphasis was given to the issue of parameter sensitivity and correlation between parameters to improve the quality of the model.

#### Time series/stimulus-response measurements on primary human hepatocytes

From the metabolite concentrations in primary human hepatocytes of individual 1 (see Additional file 3 - Atorvastatin metabolite concentrations from the time-series experiment on primary human hepatocytes of individual 1) we calculated total recovery at each time point (Table 1). The recovery is calculated from material balance equations and is defined as the sum of intracellular and extracellular metabolite amounts at the respective time-point divided by initial AS amount. As evident from Table 1, the total recovery was close to 100% after ten minutes but decreased at higher time-points. This may be explained by unspecific protein binding leading to the pool of bound metabolites, which increases over time due to intracellular accumulation of metabolites. This result points to the necessity and importance of the implementation of unspecifically bound metabolites in the model.

**Table 1:** Recovery of atorvastatin metabolites in the time-series experiment on primary human hepatocytes of Individual 1

time [min]	Recovery [%]
0	100
10	97.6
30	96.8
60	94.8
120	88.8
180	86.5
240	89.0
300	89.0
360	80.8
480	n.o.
600	78.2

The recovery, calculated from material balance equations at each experimental time-point, is defined as the sum of intracellular and extracellular metabolite amounts divided by measured initial atorvastatin acid amount (n.o.: not observed) (see Additional file 3 - Atorvastatin metabolite concentrations from the time-series experiment on primary human hepatocytes of individual 1).

#### Parameter estimation

Parameter optimizations were performed with aid of evolutionary algorithms (JavaEva, μ = 8 parent and λ = 4 children) and using the nominal parameter values of the reactions, transport and diffusion steps in equations (2) to (9). The optimization criterion in equation (10) was used, which demands to minimize the difference between experimental concentration data and model simulation. In the optimization procedure certain parameters were fixed to values (Table 2), which were either identified in or assumed from previous investigations. The Michaelis-Menten constants *K*_*M *_of the CYP3A4 hydroxylation and of the AS transporters OATP1B1 and OATP2B1 were fixed to values determined with recombinant enzymes [50,53,54]. The *K*_*M *_and *K*_*I *_of the UGT1A3-lactonization were fixed to values determined on human liver microsomes [41]. The rate constant of spontaneous hydrolysis of ASL, *k*_*CR*_, was estimated from experimental observations [43] and assumed to be same for the hydrolysis of the lactone metabolites, ASLpOH and ASLoOH, respectively. The dissociation rate constant *k*_*dis *_of unspecific binding was fixed to a value, considered to be very high in a previous study on modeling of protein binding mechanisms [33].

**Table 2:** Parameters which were fixed in the optimization procedure to literature values

Parameter	Value	Units	Literature
K_M,3A4,ASpOH_	25600	pmol·ml^-1^	[50]
K_M,3A4,ASoOH_	29700	pmol·ml^-1^	[50]
K_M,3A4,ASLpOH_	1400	pmol·ml^-1^	[50]
K_M,3A4,ASLoOH_	3900	pmol·ml^-1^	[50]
K_M,1B1,AS_	18900	pmol·ml^-1^	[54]
K_M,2B1,AS_	200	pmol·ml^-1^	[53]
K_M,1A3,AS_	12000	pmol·ml^-1^	[41]
K_I,1A3,AS_	75000	pmol·ml^-1^	[41]
k_CR_	0.0025	min^-1^	[43]
k_dis_	600	min^-1^	[33]

Parameter values were adopted from literature sources outlined in the right column.

#### Analysis of the predicted concentration-time-profiles

**Figure 2:**
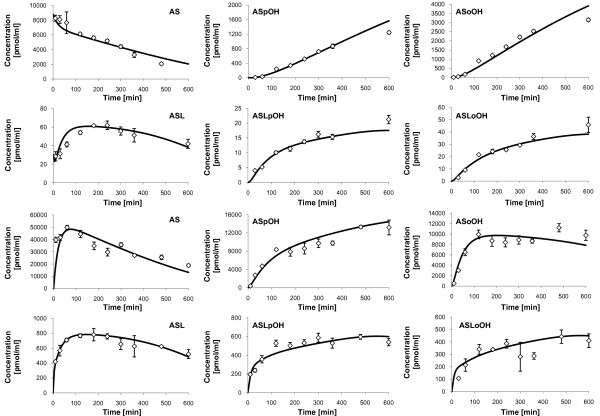
**Measured concentrations and simulated fits of Atorvastatin compounds, individual 1**. Displayed are the measured concentrations (rhombs) with mean and standard deviation (n = 3) of Atorvastatin compounds in the culture medium (upper two rows) and free Atorvastatin compounds inside primary hepatocytes (lower two rows) of individual 1, and corresponding simulation profiles (solid lines). First and third row: AS (left), ASpOH (middle) and ASoOH (right). Second and fourth row: ASL (left), ASLpOH (middle) and ASLoOH (right).

The model predicted concentration-time-profiles are illustrated together with the measured concentrations (see Additional file 3 - Atorvastatin metabolite concentrations from the time-series experiment on primary human hepatocytes of individual 1) in Figure 2. Both intracellular AS and ASL are converted to the corresponding para- and ortho-hydroxy metabolites, ASpOH and ASoOH, and ASLpOH and ASLoOH, respectively. However, the acidic metabolites, ASpOH and ASoOH, show higher intracellular concentrations than the lactone metabolites, ASLpOH and ASLoOH. The ratio between intracellular *AUC*_*0-600 min *_of the acidic form to that of the lactone form equals 20.1 in case of the para-hydroxy-metabolites and 23.6 in the case of the ortho-hydroxy-metabolites.

Accordingly, the extracellular concentrations of the acidic metabolites are higher than that of the lactone metabolites. The ratio between extracellular *AUC*_*0-600 min *_of the acidic form to that of the lactone form equals 54.3 in case of the para-hydroxy-metabolites and 70.8 in the case of the ortho-hydroxy-metabolites.

Further, the results show that the components have higher concentrations in the intracellular space than outside the cell. The ratio of intracellular *AUC*_*0-600 min *_to extracellular *AUC*_*0-600 min *_equals minimally 4.5 in the case of ASoOH and maximally 38.2 in the case of ASLpOH. Similarly, the ratio of intracellular *c*_*max *_to extracellular *c*_*max *_equals minimally 2.5 in the case of ASoOH and maximally 34.5 in the case of ASLpOH.

### Simultaneous model verification on different individual hepatocyte donors

The process of model verification so far has been applied on a single experiment on primary human hepatocytes of individual 1. In the next step it has to be proven, that the model is also capable to describe different individual metabolic profiles, especially being further able to reflect the inter-individual parameter variability, not only in the parameters *r*_*max *_of the phase I and II reactions, but also in the transporters. Therefore, the model verification is performed based on AS biotransformation data on primary hepatocytes from three different individuals simultaneously.

Therefore, maximal rate constants *r*_*max *_of CYP3A4 hydroxylation and UGT1A3 lactonization were predicted for individual 2 and 3 via the relative abundance approach (equation (11)) using the *r*_*max*__-_parameters of individual 1 (Table 3) and the protein concentrations observed on the primary human hepatocytes (Table 4). These parameters were then fixed in the optimization. Further, the rate constants *k *of PON mediated lactone hydrolysis of individual 2 and 3 were estimated in the optimization procedure.

**Table 3:** Verified parameters of CYP3A4 and UGT1A3 in individual 1

Parameter	Value	rel. Error [%]	Units
r_max,3A4,ASpOH_	1108	5.5	pmol·min^-1^·ml^-1^
r_max,3A4,ASoOH_	3345	2.3	pmol·min^-1^·ml^-1^
r_max,3A4,ASLpOH_	1228	12.7	pmol·min^-1^·ml^-1^
r_max,3A4,ASLoOH_	2756	11.6	pmol·min^-1^·ml^-1^
r_max,1A3,AS_	956	14.8	pmol·min^-1^·ml^-1^

Parameters are listed with nominal values from optimization and relative parameter errors from **FIM **based identifiability analysis (see Additional file 4 - Analysis of parameter sensitivity and following model reduction procedure) in the corresponding units shown.

**Table 4:** Protein concentrations of CYP3A4 and UGT1A3 in primary human hepatocytes

Individual	**CYP3A4 [pmol ml**^**-1**^**]**		UGT1A3 [-]
1	1027	± 107 (n = 2)	1.00
2	611	± 120 (n = 4)	0.29
3	755	± 19 (n = 2)	0.10

Protein concentrations were calculated from total CYP3A4 and UGT1A3 content per sample divided by total intracellular volume of hepatocytes, respectively. Protein concentrations of CYP3A4 are listed in mean and standard deviation in the units pmol per ml intracellular volume. Relative protein concentrations of UGT1A3 are normalized to individual 1.

**Figure 3:**
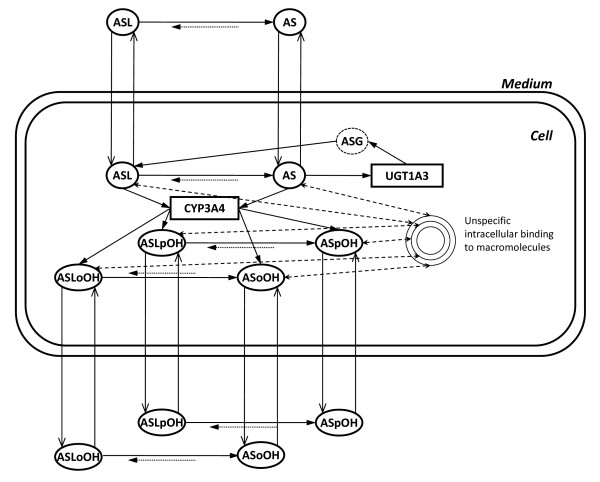
**Scheme of the model used in the simultaneous model verification**. In contrast to the full model illustrated in Figure 1, this model contains lumped transport kinetics, which means, one import and one export rate for each metabolite, respectively. This transport simplification resulted from the local parameter sensitivity analysis (see Additional file 4 - Analysis of parameter sensitivity and following model reduction procedure).

However, the analysis of parameter sensitivity and identifiability (see Additional file 4 - Analysis of parameter sensitivity and following model reduction procedure) showed that the first order kinetics of OATP1B1 mediated import is favored over the zero-order kinetics of OATP2B1 import of AS, and that the first order kinetics are hard to distinguish from first order passive diffusion mechanisms in this evaluation system. Thus, in the following, mechanisms of active transport and passive diffusion are lumped, resulting in the apparent transport rate for import,(12)

and in the apparent transport rate for export(13)

described by the product of apparent rate constant *κ*_*im/ex *_of import or export and extra- or intracellular concentration , respectively (Figure 3). Consequently, the rate constants *κ *of import and export are set individually, and were to be estimated in the optimization procedure.

The simultaneous model verification was performed based on the stimulus response data obtained from primary human hepatocytes of individual 1 (see Additional file 3 - Atorvastatin metabolite concentrations from the time-series experiment on primary human hepatocytes of individual 1), individual 2 (see Additional file 5 - Atorvastatin metabolite concentrations from the time-series experiment on primary human hepatocytes of individual 2) and individual 3 (see Additional file 6 - Atorvastatin metabolite concentrations from the time-series experiment on primary human hepatocytes of individual 3), respectively.

**Figure 4:**
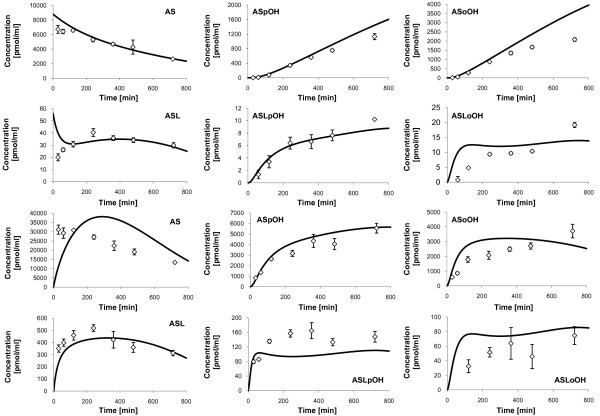
**Measured concentrations and simulated fits of Atorvastatin compounds, individual 2**. Displayed are the measured concentrations (rhombs) with mean and standard deviation (n = 3) of Atorvastatin compounds in the culture medium (upper two rows) and free Atorvastatin compounds inside primary hepatocytes (lower two rows) of individual 2, and corresponding simulation profiles (solid lines). First and third row: AS (left), ASpOH (middle) and ASoOH (right). Second and fourth row: ASL (left), ASLpOH (middle) and ASLoOH (right).

**Figure 5:**
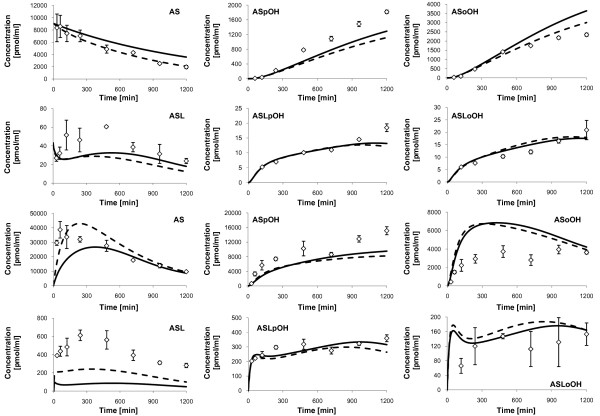
**Measured concentrations and simulated fits of Atorvastatin compounds, individual 3**. Displayed are the measured concentrations (rhombs) with mean and standard deviation (n = 3) of Atorvastatin compounds in the culture medium (upper two rows) and free Atorvastatin compounds inside primary hepatocytes (lower two rows) of individual 3, and corresponding simulation profiles (solid lines and dashed lines). First and third row: AS (left), ASpOH (middle) and ASoOH (right). Second and fourth row: ASL (left), ASLpOH (middle) and ASLoOH (right). The dashed lines show the additional consideration of putative beta-oxidation of atorvastatin acids, AS, ASpOH and ASoOH, and of adaptation of CYP3A4 and UGT1A3 protein concentrations in the model fit.

The model prediction is in satisfying agreement with experimental data of individual 2 (Figure 4). However, in case of individual 3, the model prediction is comparably poor, because there are relatively high deviations, especially with the intracellular metabolites ASL and ASoOH and extracellular ASpOH (Figure 5, solid line). One reason could be the extended contribution of beta-oxidation in AS metabolism. Beta oxidation at the heptanoic acid side chain is a typical transformation pathway for all statins, but is reported to play only a minor role in humans [75]. However, high activity of beta-oxidation of fatty acids, which is responsible for the supply of ATP for gluconeogenesis in type 2 diabetes mellitus [76,77], which individual 3 was diagnosed with, may severely influence the AS metabolism. To test this hypothesis, respective reactions with the acid metabolites as substrates were considered in the model verification of individual 3, but the results showed no significant improvement (data not shown). The second reason could be, that CYP3A4 and UGT1A3 protein concentrations of individual 3 used in the estimation of corresponding *r*_*max *_value via relative abundance approach (equation (11)) differ from the measured mean value (Table 4). Due to the fact that the *r*_*max *_parameters of CYP3A4 and UGT1A3 show high sensitivities in the parameter sensitivity analysis (see Additional file 4 - Analysis of parameter sensitivity and following model reduction procedure), a variation in the values would have a high impact on the metabolic profiles. Therefore, in a further optimization step, the CYP3A4 protein concentrations are allowed to vary in the interval of mean ± standard deviation (Table 4), where the standard deviation of UGT1A3 protein concentration is assumed to be 30% of the mean value. Notably, an improvement in the model prediction on individual 3 could be achieved in the case of the intracellular metabolites AS and ASL and extracellular AS (Figure 5, dashed lines). But there are still major deviations in case of intracellular ASL and ASoOH and extracellular ASpOH, which could not be explained any further, but shows, that there must be some other reactions or effects in the system, which have not been discovered yet.

The estimated model parameters for individual 1, 2 and 3, summarized in Table 5, display the proposed parameter variability of enzyme mediated reactions as well as of the transport steps. However, the rate constants of ASLoOH import and export of individual 2 and of ASL import and export of individual 3 are considerably higher than the other transport rate constants, which points to strong linear dependencies of these parameters.

**Table 5:** Parameter Variability in the simultaneous model verification on individual atorvastatin metabolism data.

Parameter	Individual 1	Individual 2	Individual 3	Units
r_max,3A4,ASpOH_	1108	660	606	pmol·min^-1^·ml^-1^
r_max,3A4,ASoOH_	3345	1991	1830	pmol·min^-1^·ml^-1^
r_max,3A4,ASLpOH_	1228	731	672	pmol·min^-1^·ml^-1^
r_max,3A4,ASLoOH_	2756	1640	1508	pmol·min^-1^·ml^-1^
r_max,1A3,AS_	957	281	120	pmol·min^-1^·ml^-1^
κ_PON,ASL_	308	99.2	0.00	10^-3 ^min^-1^
κ_PON,ASLOH_	280	545	82.9	10^-3 ^min^-1^

κ_ex,AS_	2.17	0.16	0.17	μL·min^-1^
κ_ex,ASL_	21.8	2.48	10.5	μL·min^-1^
κ_ex,ASpOH,_	0.80	1.25	0.52	μL·min^-1^
κ_ex,ASoOH_	1.60	3.62	0.91	μL·min^-1^
κ_ex,ASLpOH_	1.13	1.01	0.59	μL·min^-1^
κ_ex,ASLoOH_	2.67	86.3	0.77	μL·min^-1^
κ_im.AS_	20.3	4.42	3.45	μL·min^-1^
κ_im.ASL_	275	25.8	82.7	μL·min^-1^
κ_im,ASpOH_	3.96	2.01	2.53	μL·min^-1^
κ_im,ASoOH_	0.39	0.22	0.00	μL·min^-1^
κ_ex,ASLpOH_	33.7	7.24	8.59	μL·min^-1^
κ_im,ASLoOH_	26.1	530	2.43	μL·min^-1^

k_B-Ox,AS_			15.0	10^-3 ^min^-1^
k_B-Ox,ASOH_			0.00	10^-3 ^min^-1^

Parameters are listed for the individuals 1, 2 and 3 with nominal values from optimization in the corresponding units shown.

The mathematical model of AS metabolism in human hepatocytes of individual 1 is supplemented as SBML-file (see Additional file 7 - Model of atorvastatin metabolism in primary human hepatocytes of individual 1, and BioModels database).

### Dynamic analysis of inter-individual CYP3A4 and UGT1A3 expression level variability

**Figure 6:**
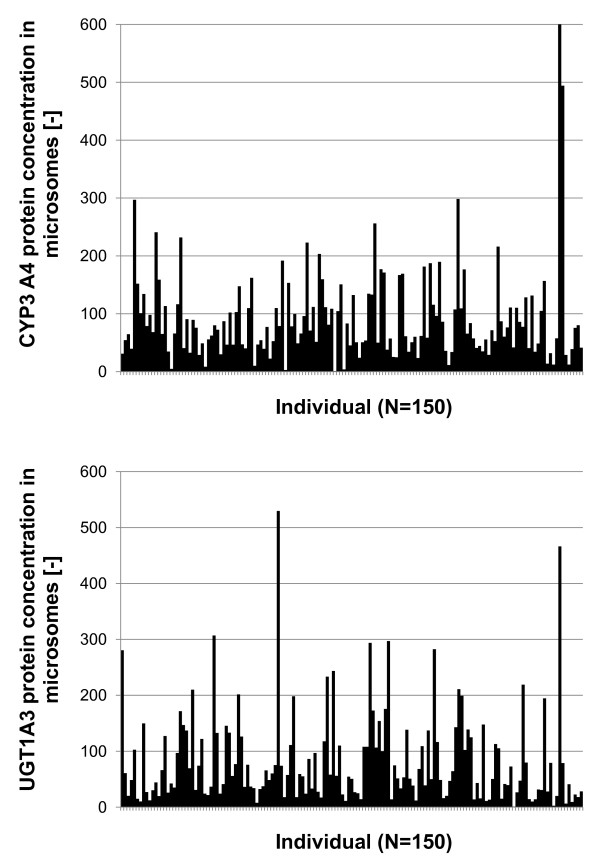
**Individual variability of CYP3A4 and UGT1A3 protein concentration level**. Displayed is the distribution of CYP3A4 (top) and UGT1A3 (bottom) protein concentration data of individual human liver microsomes (n = 150). Protein concentrations are normalized to minimal value, respectively.

Based on the model version of optimized parameters, gained in the simultaneous model fit, the effect of inter-individual variability of CYP3A4 and UGT1A3 protein expression levels was investigated by linking the protein expression data of 150 liver samples (Figure 6) via the described relative abundance approach in equation (11), using individual 1 as reference. UGT1A3 and CYP3A4 protein concentrations of individual 1 were converted from based on total protein amount to based on microsomal protein amount by multiplying with the factor 0.22, determined in human liver homogenates and corresponding microsome fractions via Bradford test [78].

Simulations were performed with an initial extracellular AS concentration of 50 (pmol ml^-1^), which is in the range of physiological plasma concentrations [54,79], over a time period of 1200 min.

**Figure 7:**
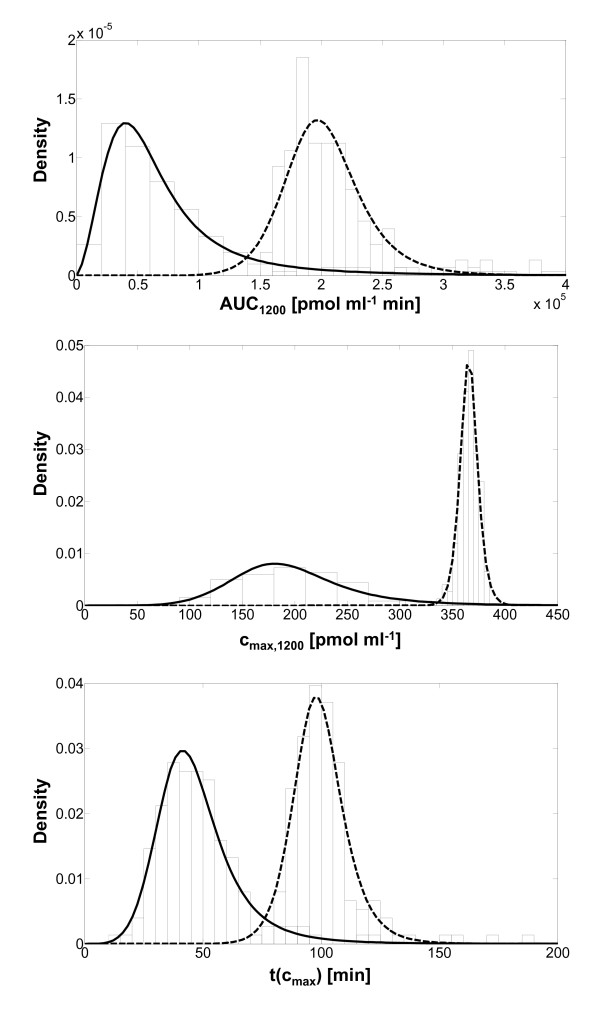
**Distributions from dynamic analysis of the Inter-individual variability in Atorvastatin metabolism**. Distributions (bars) of AUC (top), c_max _(middle) and of t(c_max_) (bottom), and fitted probability density functions of simulated profiles of AS (solid lines) and of the sum of AS, ASpOH and ASoOH (dashed lines), respectively. Distributions are predicted by the dynamic simulation of Atorvastatin metabolism, implementing individual CYP3A4 and UGT1A3 protein concentration data (**Figure 6**).

The most important question that arises from this dynamic analysis was, how the metabolic profiles of the intracellular metabolites AS, ASpOH and ASoOH are influenced by this variability, since they are considered to be the active drugs, which inhibit HMGCoA-reductase [80]. Therefore, AUC, *c*_*max *_and *t(c*_*max*_*) *of the concentration-time-profiles of either AS alone or the sum of concentration of AS, ASpOH and ASoOH were calculated for each liver sample over a time period of 1200 min and the distributions over all liver samples were evaluated, respectively. Finally, appropriate probability density functions are fitted to the distributions (Figure 7). The probability density function characteristics are summarized in Table 6. Obviously, there are differences between the examination of only AS or the sum of concentrations of AS, ASpOH and ASoOH. AUC, *c*_*max *_and *t(c*_*max*_*) *have lower values in case of AS alone compared to the sum of all acidic metabolites. The population mean of AUC is 75051 (pmol ml^-1 ^min) in the case of AS and 203617 (pmol ml^-1 ^min) in the case of the sum of AS, ASpOH and ASoOH. Also, c_max _is lower in the case of AS alone, 201 (pmol ml^-1^), compared to the sum of the acidic metabolites, 366 (pmol ml^-1^). Further, the maximal concentration appears at a shorter time point, 48 min, in the case of AS alone, compared to the time point, 100 min, of the sum of AS, ASpOH and ASoOH. The results are quite explainable, since ASpOH and ASoOH are the hydroxylated products of AS and therefore their maximal concentrations event at a delayed time-point compared to AS.

**Table 6:** Probability density functions fitted to inter-individual variability distributions

	Probability density function	Mean	**s.d**.	rel. s.d. [%]
**AUC [pmol ml**^-1^*min]				
AS	Log-logistic	75051	92038	123
AS+ASpOH+ASoOH	Log-logistic	203617	35775	18

**c**_**max **_**[pmol ml**^**-1**^**]**				
AS	Log-logistic	201	64	32
AS+ASpOH+ASoOH	Log-logistic	366	10	3

**t(c**_**max**_**) [min]**				
AS	Log-logistic	48	18	39
AS+ASpOH+ASoOH	Log-logistic	100	12	12

Probability density functions fitted to distributions of AUC, c_max _and t(c_max_) (depicted in **Figure 7**), generated in dynamic simulations considering inter-individual variability of CYP3A4 and UGT1A3 protein concentration data (**Figure 6**). Distributions of AUC, c_max _and t(c_max_) were fitted best with Log-logistic function. Displayed are the mean, the standard deviation (s.d.), and the relative standard deviation (rel. s.d.) of the fitted function, respectively.

Further, except for the AUC of AS, the probability density functions show a very narrow shape, regarding the relative standard deviation, when comparing to the sample standard deviations of the underlying distribution of CYP3A4 and UGT1A3, respectively. The probability density functions fitted to CYP3A4 and UGT1A3 distributions have a relative standard deviation of 259% and 137%, respectively, whereas the relative standard deviations of the probability density functions of AUC, c_max _and t(c_max_) are lower than 50%, except for the probability density function of AUC of intracellular AS, which equals 123%.

## Discussion

The deterministic modeling of drug metabolism has a major advantage compared to traditional pharmacokinetic models. The distinction between metabolism and elimination inside the hepatocytes and the detailed description of the biotransformation network structure allows the observation of the reaction kinetics and the transport mechanisms involved. Our modeling considered the major acid and lactone metabolites in the extracellular and intracellular space as well as appropriate reaction kinetics and transport steps. An experimental limitation in primary hepatocytes concerns the lower limit of quantification of each metabolite. Therefore, a relatively high initial concentration of AS of about 10 μM had to be chosen in order to permit measurement of the described major metabolites. Interestingly, the intracellular concentration of the parent drug AS was higher than the extracellular concentration. This indicates, that either the OATP mediated import does rather follow an active uptake mechanism than the proposed facilitated diffusion mechanism [58,59], or so far unknown transporters may be involved in the transport steps. By any means it justifies the chosen implementation of kinetics for the active mediated transport beside the passive diffusion mechanism. In contrast to concentrations determined in plasma in clinical studies [54,79,81], extracellular concentrations of the acidic metabolites were much higher compared to the lactones. A possible explanation of these discrepancies may be that the *in-vitro *investigation on isolated primary human hepatocytes was performed over rather short time intervals (hours), whereas analyses in humans usually cover longer periods (days). A second explanation could be the relatively high initial concentration of AS of about 10 μM used in this study, compared to plasma concentrations observed to be lower than 200 (pmol ml^-1^). Furthermore, we found that the recovery of metabolites decreases over the time. This confirms on the one hand the contribution of unspecific binding to macromolecules, most severely in the intracellular space, as observed previously [35], but on the other hand could also be contributed to a certain extent by the unspecific binding to the collagen layer or the plates [34], especially of highly lipophilic ASL and lactone metabolites. However, due to the washing procedure preliminary to the cell harvesting and disruption procedure, this effect could not be observed in the chosen experimental set-up.

The parameter identification was considered satisfactory, as the simulation profiles fitted well to the corresponding measured metabolite concentration data. But, it was questionable if the optimized parameters could be considered to be sensitive and identifiable. Therefore, a local parameter sensitivity and identifiability analysis based on the Fisher-Information-Matrix was performed. The results showed that the full model of AS metabolism is not identifiable. Consequently a model reduction procedure was set-up and could be applied successfully, indicating that the parameter identification difficulties are caused by non-identifiable transporter steps, displayed by low parameter sensitivities and high parameter errors and correlations, which were then reduced in the model. However, this outcome does not necessarily mean that the non-identifiable transport steps are not present or not used in human hepatocytes, but rather implies, that they cannot be distinguished from the remaining transport steps in the model verification. Thus, the remaining transport steps in the model capture the probable superimposed contribution of several transport mechanisms *in-vivo*.

Unfortunately, the local identifiability of the final model version reveals some uncertainties caused by remaining high correlations present in the transport steps, as well as in the intracellular reaction network. An additional problem attributed to the modeling and parameter optimization is related to the fact that several parameters have lumped characteristics, because they are used for more than one compound, like the fraction unbound factor, which is set to be the same for all acidic or lactone AS compounds, respectively. A possible solution would be to set individual parameters for each compound. However, then the parameter identifiability difficulties would be even more severe. This is due to the fact, that the experimental observation on primary human hepatocytes is constrained strongly on several limitations. For example, the range of initial concentrations is strongly limited to the quantification of metabolites of interest. Further, the number of data points is strongly limited to the available cell number of primary hepatocytes, isolated from surgery removal. Finally, also the individual character of human cells due to individual genetic and medical background precludes an exact reproduction of experiments and the reproducibility of experimental results. The remaining parameter uncertainty seems to be crucial for the prediction of the population probability on the first sight. Actually, this is not the case. Since the parameter errors and the correlations indicate which parameter changes in what extent do not influence the computed time courses, the variability in the respective parameter error range would not influence the probability distributions. In general, the question if parameter errors and insensitivities are sufficiently small can only be answered when knowing the requirements of the pharmacological application as drug, which was not given in this study.

The simultaneous model verification based on primary human hepatocytes from different individuals illustrates the individual character of drug metabolism in the liver. Further, it indicates the inter-individual variability of rate parameters not only of the phase I and II enzymes, but also of the transport enzymes. In case of patients with type 2 diabetes, AS metabolism is probably influenced strongly by beta-oxidation and further so far unknown effects. Therefore, the individual model verification might also be used to reveal undesired pharmacokinetic effects. However, this analysis tool should be checked carefully on other drug systems in the future, too.

The individual nature of the AS metabolism was investigated in this study in the domain of inter-individual variability of CYP3A4 and UGT1A3 protein expression levels in human hepatocytes, by performing dynamic analysis on the verified model, individualized by implementation of a comprehensive set of protein data from a liver bank. The results show, that the inter-individual variability strongly affects the biotransformation behavior and therefore reveals the individual character of AS metabolism. Consequently, this individuality in the pharmacokinetics of AS also points out the individual character of pharmacodynamics at the drug target, namely the HMGCoA-reductase in human hepatocytes.

However, this subject-variability can also be expected to be present in the transport protein expression, as previously shown for OATP-C [82]. Therefore, this variability should be taken into account in the future by implementing corresponding population expression data in the dynamic analysis. Another source of variation is the well-known polymorphism in CYP- and UGT- enzymes and likely transport proteins, which very probably causes inter-individual variations of the catalytic activity and the substrate affinity *K*_*M*_. Thus, further efforts should also consider this individual difference, leading to the improvement of the model prediction.

To resolve the contributing transport mechanisms in more detail, additional information should be implemented from transport investigations on recombinant systems, as described previously [72,83], which enable the differentiated identification of both basolateral and apical transporters of AS and the corresponding transport kinetics. Further, also the estimation of unspecific drug protein binding could be improved in the future by using radiolabeled compounds in the experiments and modeling approaches as described previously [60,69].

## Conclusions

We believe that the results of our simulations provide strong arguments for rigorous dynamic modeling of drug biotransformation at the cellular level embedded in a systems biology approach. By resolving the detailed metabolic network structure with metabolites and catalyzing enzymes, we investigated the dynamic variation of atorvastatin metabolism affected by the inter-individual variability of expression levels of phase I and phase II enzymes.

In contrast to experimental investigations on recombinant systems or tissue fractions of hepatocytes, like microsomes, the investigation on primary human hepatocytes enabled the holistic and most realistic in-vitro observation of drug biotransformation, because it is possible to observe the coupled contribution of metabolism and transport to the entire processes. De novo of this study, we identified intracellular concentration profiles of atorvastatin metabolites in primary human hepatocytes in a time-series approach.

Such an approach is essential for integration of further-reaching issues, such as drug-drug interactions, impact of regulation networks linked to nuclear receptors and particularly to quantitatively account for subject-variability. The integration of this variability caused by genetic or environmental variations is crucial for predictive pharmacokinetic modeling.

Such a rigorous modeling approach critically depends upon a tight link between experimental observations and model design, simulation and verification. While the results are promising, some limitations in the parameter identification were still encountered. In the long term, these open problems can only be solved by stronger links to other research areas, such as pharmacogenetics, characterization of transporters, etc. On the whole, our contention is that the problem of parameter identifiability is an indispensable ingredient of model verification. Systematic investigations - if possible linked to optimal experimental design - can greatly strengthen the credibility of the models.

However, we present a model that goes much further. The domain of application does not remain the system behavior for which it has been elaborated. The model provides the possibility to link further modules such as gene regulation, drug target metabolism and present the important links to be implemented into the PBPK environment. Finally, the model structure used in this study should be considered as a module to be integrated into the framework of multi-scale whole body modeling and simulations necessary to tackle the drug disposition in patient populations.

## List of abbreviations

AS: atorvastatin acid; ASL: atorvastatin lactone; ASOH: hydroxy-atorvastatin acids (para- and ortho-); ASpOH: para-hydroxy-atorvastatin acid; ASoOH:ortho-hydroxy-atorvastatin acid; ASLOH: hydroxy-atorvastatin lactones (para- and ortho-); ASLpOH: para-hydroxy-atorvastatin lactone; ASLoOH: ortho-hydroxy-atorvastatin lactone; B-OX: beta-oxidation of atorvastatin acids; *c: *index: intracellular (cytosol); *calc: *index: calculated; *CR: *index: chemical reaction of hydrolysis of lactones; CYP: cytochrome P450 monooxygenase; *Den: *Denominator; *e: *index: enzyme; *eq: *Index: equilibrium; *ex: *index: export; FIM: Fisher-Information-Matrix; *fu: *fraction unbound; *i: *index: reaction; *im: *index: import; *j*: *J; *index: compound; *k: *index: parameter; rate constant; *K*_*M*_: Michaelis-Menten constant; *l: *index: parameter; *m: *index: extracellular (medium); meas: index: measured; n:N; index: time-point; MDR: multidrug resistance protein; MRP: multidrug resistance-related protein; OATP: organic anion transport protein; PON: paraoxanase; r: reaction rate; rmax: maximal reaction rate; UGT: UDP-glucuronosyl-transferase; 1A3: index: UGT1A3; 1B1: index: OATP1B1; 2B1: index: OATP2B1; 3A4: index: CYP3A4;

## Authors' contributions

JB carried out the experiments on primary human hepatocytes, set-up the model of atorvastatin metabolism, performed the model verification and dynamic analysis, and drafted the manuscript. SR performed the quantification of metabolites. SR and AS performed the quantification of protein data, and contributed to the experimental design and the manuscript draft. GV guided the culturing of primary human hepatocytes at the Institute of Biochemical Engineering and assisted in the conduction of the experiments. KM contributed to the project design and guided the protein quantification. AKN, TSW and WET provided primary human hepatocytes. UZ and MR contributed to the design of the project and the fund raising, guided the research, and revised the manuscript draft critically. UZ further provided individual liver protein data. MR was the coordinator of the project. All authors read and approved the final manuscript.

## Supplementary Material

Additional file 1 **Derivation of atorvastatin kinetics at CYP3A4**. This file contains the derivation of the atorvastatin kinetics at CYP3A4, which describes the competition of alternative substrate degradation to alternative products (supplemented as .pdf-file). Click here for file

Additional file 2 **Derivation of kinetics of unspecific binding to macromolecules**. This file contains the derivation of kinetics of the unspecific binding of atorvastatin metabolites to macromolecules, for example proteins (supplemented as .pdf-file). Click here for file

Additional file 3 **Atorvastatin metabolite concentrations from the time-series experiment on primary human hepatocytes of individual 1**. Extracellular concentrations (upper part) and intracellular concentrations (lower part) of atorvastatin acid and lactone (AS and ASL) and corresponding para- and ortho-hydroxy-metabolites (acids: ASpOH and ASoOH; lactones: ASLpOH and ASLoOH) at the defined time-points with mean and standard deviation (n = 3) from triplicate measurements per LC-MS/MS (n.d.: not determinable; n.o.: not observed). The recovery is calculated from material balance equations and is defined as the sum of intracellular and extracellular metabolite amounts at the respective time-point divided by initial AS amount (supplemented as .pdf-file). Click here for file

Additional file 4 **Analysis of parameter sensitivity and following model reduction procedure**. This file contains the analysis of parameter sensitivity and the exemplary model reduction procedure, which is necessary for achieving a high quality predictive model of atorvastatin metabolism (supplemented as .pdf-file). Click here for file

Additional file 5 **Atorvastatin metabolite concentrations from the time-series experiment on primary human hepatocytes of individual 2**. Extracellular concentrations (upper part) and intracellular concentrations (lower part) of atorvastatin acid and lactone (AS and ASL) and corresponding para- and ortho-hydroxy-metabolites (acids: ASpOH and ASoOH; lactones: ASLpOH and ASLoOH) at the defined time-points with mean and standard deviation (n = 3) from triplicate measurements per LC-MS/MS (n.d.: not determinable) (supplemented as .pdf-file). Click here for file

Additional file 6 **Atorvastatin metabolite concentrations from the time-series experiment on primary human hepatocytes of individual 3**. Extracellular concentrations (upper part) and intracellular concentrations (lower part) of atorvastatin acid and lactone (AS and ASL) and corresponding para- and ortho-hydroxy-metabolites (acids: ASpOH and ASoOH; lactones: ASLpOH and ASLoOH) at the defined time-points with mean and standard deviation (n = 3) from triplicate measurements per LC-MS/MS (n.d.: not determinable) (supplemented as .pdf-file). Click here for file

Additional file 7 **Model of atorvastatin metabolism in primary human hepatocytes of individual 1**. The sbml-file contains the model of Atorvastatin metabolism in human hepatocytes with the model parameters identified on primary human hepatocytes of individual 1 (supplemented as .xml-file). Click here for file

## References

[B1] Bugrim A Nikolskaya T Nikolsky Y Early prediction of drug metabolism and toxicity: systems biology approach and modeling Drug Discovery Today 2004 5 127 135 10.1016/S1359-6446(03)02971-4 14960390

[B2] van de Waterbeemd H Gifford E ADMET in silico modelling: Towards prediction paradise? Nature Reviews Drug Discovery 2003 5 192 204 10.1038/nrd1032 12612645

[B3] Kramer JA Sagartz JE Morris DL The application of discovery toxicology and pathology towards the design of safer pharmaceutical lead candidates Nat Rev Drug Discov 2007 5 636 649 10.1038/nrd2378 17643090

[B4] Huisinga W Telgmann R Wulkow M The virtual laboratory approach to pharmacokinetics: design principles and concepts Drug Discovery Today 2006 5 800 805 10.1016/j.drudis.2006.07.001 16935747

[B5] Kitano H Systems biology: a brief overview Science 2002 5 1662 1664 10.1126/science.1069492 11872829

[B6] Spivey A Systems biology - The big picture Environmental Health Perspectives 2004 5 A938 A943 15598608 10.1289/ehp.112-a938 PMC1247669

[B7] Andersson TB Bredberg E Ericsson H Sjoberg H An evaluation of the in vitro metabolism data for predicting the clearance and drug-drug interaction potential of CYP2C9 substrates Drug Metabolism and Disposition 2004 5 715 721 10.1124/dmd.32.7.715 15205386

[B8] Clarke SE Jeffrey P Utility of metabolic stability screening: comparison of in vitro and in vivo clearance Xenobiotica 2001 5 591 598 10.1080/00498250110057350 11569527

[B9] Lin JH Sense and nonsense in the prediction of drug-drug interactions Current Drug Metabolism 2000 5 305 331 10.2174/1389200003338947 11465042

[B10] de Groot MJ Ekins S Pharmacophore modeling of cytochromes P450 Advanced Drug Delivery Reviews 2002 5 367 383 10.1016/S0169-409X(02)00009-1 11922953

[B11] Gasteiger J Reitz M Han YQ Sacher O Analyzing biochemical pathways using neural networks and genetic algorithms Australian Journal of Chemistry 2006 5 854 858 10.1071/CH06140

[B12] Korolev D Balakin KV Nikolsky Y Kirillov E Ivanenkov YA Savchuk NP Ivashchenko AA Nikolskaya T Modeling of human cytochrome P450-mediated drug metabolism using unsupervised machine learning approach Journal of Medicinal Chemistry 2003 5 3631 3643 10.1021/jm030102a 12904067

[B13] Lewis DFV Ioannides C Parke DV An improved and updated version of the compact procedure for the evaluation of P450-mediated chemical activation Drug Metabolism Reviews 1998 5 709 737 10.3109/03602539808996328 9844807

[B14] Lewis DFV Ioannides C Parke DV Further evaluation of COMPACT, the molecular orbital approach for the prospective safety evaluation of chemicals Mutation Research-Genetic Toxicology and Environmental Mutagenesis 1998 5 41 54 10.1016/S1383-5718(97)00145-9 9508363

[B15] Terfloth L Bienfait B Gasteiger J Ligand-based models for the isoform specificity of cytochrome P450 3A4, 2D6, and 2C9 substrates Journal of Chemical Information and Modeling 2007 5 1688 1701 10.1021/ci700010t 17608404

[B16] Zhang EY Phelps MA Cheng C Ekins S Swaan PW Modeling of active transport systems Advanced Drug Delivery Reviews 2002 5 329 354 10.1016/S0169-409X(02)00007-8 11922951

[B17] Ekins S Erickson JA A pharmacophore for human pregnane X receptor ligands Drug Metabolism and Disposition 2002 5 96 99 10.1124/dmd.30.1.96 11744617

[B18] Ekins S Mirny L Schuetz EG A ligand-based approach to understanding selectivity of nuclear hormone receptors PXR, CAR, FXR, LXR alpha, and LXR beta Pharmaceutical Research 2002 5 1788 1800 10.1023/A:1021429105173 12523656

[B19] Hou TJ Wang JM Li YY ADME evaluation in drug discovery. 8. The prediction of human intestinal absorption by a support vector machine Journal of Chemical Information and Modeling 2007 5 2408 2415 10.1021/ci7002076 17929911

[B20] Hou TJ Wang JM Zhang W Xu XJ ADME evaluation in drug discovery. 6. Can oral bioavailability in humans be effectively predicted by simple molecular property-based rules? Journal of Chemical Information and Modeling 2007 5 460 463 10.1021/ci6003515 17381169

[B21] Hou TJ Zhang W Xia K Qiao XB Xu XJ ADME evaluation in drug discovery. 5. Correlation of Caco-2 permeation with simple molecular properties Journal of Chemical Information and Computer Sciences 2004 5 1585 1600 15446816 10.1021/ci049884m

[B22] Leonard JT Roy K On selection of training and test sets for the development of predictive QSAR models Qsar & Combinatorial Science 2006 5 235 251 10.1002/qsar.20051016121562827

[B23] Rostami-Hodjegan A Tucker GT Simulation and prediction of in vivo drug metabolism in human populations from in vitro data Nature Reviews Drug Discovery 2007 5 140 148 10.1038/nrd2173 17268485

[B24] Andersen ME Development of Physiologically-Based Pharmacokinetic and Physiologically-Based Pharmacodynamic Models for Applications in Toxicology and Risk Assessment Toxicology Letters 1995 5 35 44 10.1016/0378-4274(95)03355-O 7570672

[B25] Gerlowski LE Jain RK Physiologically Based Pharmacokinetic Modeling - Principles and Applications Journal of Pharmaceutical Sciences 1983 5 1103 1127 10.1002/jps.2600721003 6358460

[B26] Reddy M Yang RS Andersen ME Clewell I H J Phyiologically Based Pharmacokinetic Modelling 2005 John Wiley & Sons

[B27] Thompson CM Sonawane B Barton HA DeWoskin RS Lipscomb JC Schlosser P Chiu WA Krishnan K Approaches for applications of physiologically based pharmacokinetic models in risk assessment Journal of Toxicology and Environmental Health-Part B-Critical Reviews 2008 5 519 547 10.1080/10937400701724337 18584453

[B28] Willmann S Lippert J Sevestre M Solodenko J Fois F Schmitt W PK-Sim^®^: a physiologically based pharmacokinetic 'whole-body' model Biosilico 2003 5

[B29] Balani SK Miwa GT Gan LS Wu JT Lee FW Strategy of utilizing in vitro and in vivo ADME tools for lead optimization and drug candidate selection Current Topics in Medicinal Chemistry 2005 5 1033 1038 10.2174/156802605774297038 16181128

[B30] Hop CECA Cole MJ Davidson RE Duignan DB Federico J Janiszewski JS Jenkins K Krueger S Lebowitz R Liston TE Mitchell W Snyder M Steyn SJ Soglia JR Taylor C Troutman MD Umland J West M Whalen KM Zelesky V Zhao SX High Throughput ADME Screening: Practical Considerations, Impact on the Portfolio and Enabler of In Silico ADME Models Current Drug Metabolism 2008 5 847 853 10.2174/138920008786485092 18991580

[B31] Singh SS Preclinical pharmacokinetics: An approach towards safer and efficacious drugs Current Drug Metabolism 2006 5 165 182 10.2174/138920006775541552 16472106

[B32] Nestorov I Whole-body physiologically based pharmacokinetic models Expert Opinion on Drug Metabolism & Toxicology 2007 5 235 249 10.1517/17425255.3.2.235 17428153

[B33] Baker M Parton T Kinetic determinants of hepatic clearance: plasma protein binding and hepatic uptake Xenobiotica 2007 5 1110 1134 10.1080/00498250701658296 17968739

[B34] Poirier A Lave T Portmann R Brun ME Senner F Kansy M Grimm HP Funk C Design, Data Analysis, and Simulation of in Vitro Drug Transport Kinetic Experiments Using a Mechanistic in Vitro Model Drug Metabolism and Disposition 2008 5 2434 2444 10.1124/dmd.108.020750 18809732

[B35] Paine SW Parker AJ Gardiner P Webborn PJH Riley RJ Prediction of the pharmacokinetics of atorvastatin, cerivastatin, and indomethacin using kinetic models applied to isolated rat hepatocytes Drug Metabolism and Disposition 2008 5 1365 1374 10.1124/dmd.107.019455 18426955

[B36] Thasler WE Weiss TS Schillhorn K Stoll PT Irrgang B Jauch KW Charitable State-Controlled Foundation Human Tissue and Cell Research: Ethic and Legal Aspects in the Supply of Surgically Removed Human Tissue For Research in the Academic and Commercial Sector in Germany Cell Tissue Bank 2003 5 49 56 10.1023/A:1026392429112 15256870

[B37] Nussler AK Nussler NC Merk V Brulport M Schormann W Yao P Hengstler JG Santin M The Holy grail of hepatocyte culturing and therapeutic use Strategies in Regenerative Medicine 2008 New York: Springer

[B38] Riedmaier S Klein K Hofmann U Keskitalo JE Neuvonen PJ Schwab M Niemi M Zanger UM UDP-glucuronosyltransferase (UGT) polymorphisms affect atorvastatin lactonization in vitro and in vivo Clin Pharmacol Ther 2009 5 65 73 19794410 10.1038/clpt.2009.181

[B39] Wolbold R Klein K Burk O Nussler AK Neuhaus P Eichelbaum M Schwab M Zanger UM Sex is a major determinant of CYP3A4 expression in human liver Hepatology 2003 5 978 988 14512885 10.1053/jhep.2003.50393

[B40] Langenfeld E Zanger UM Jung K Meyer HE Marcus K Mass spectrometry-based absolute quantification of microsomal cytochrome P450 2D6 in human liver Proteomics 2009 5 2313 2323 10.1002/pmic.200800680 19402041

[B41] Goosen TC Bauman JN Davis JA Yu C Hurst SI Williams JA Loi CM Atorvastatin glucuronidation is minimally and nonselectively inhibited by the fibrates gemfibrozil, fenofibrate, and fenofibric acid Drug Metab Dispos 2007 5 1315 1324 10.1124/dmd.107.015230 17470524

[B42] Prueksaritanont T Subramanian R Fang X Ma B Qiu Y Lin JH Pearson PG Baillie TA Glucuronidation of statins in animals and humans: a novel mechanism of statin lactonization Drug Metab Dispos 2002 5 505 512 10.1124/dmd.30.5.505 11950779

[B43] Kearney AS Crawford LF Mehta SC Radebaugh GW The interconversion kinetics, equilibrium, and solubilities of the lactone and hydroxyacid forms of the HMG-CoA reductase inhibitor, CI-981 Pharm Res 1993 5 1461 1465 10.1023/A:1018923325359 8272408

[B44] Aviram M Rosenblat M Paraoxonases (PON1, PON2, PON3) analyses in vitro and in vivo in relation to cardiovascular diseases Methods Mol Biol 2008 5 259 276 10.1007/978-1-60327-517-0_20 19082953

[B45] Draganov DI Stetson PL Watson CE Billecke SS La Du BN Rabbit serum paraoxonase 3 (PON3) is a high density lipoprotein-associated lactonase and protects low density lipoprotein against oxidation J Biol Chem 2000 5 33435 33442 10.1074/jbc.M004543200 10931838

[B46] Draganov DI Teiber JF Speelman A Osawa Y Sunahara R La Du BN Human paraoxonases (PON1, PON2, and PON3) are lactonases with overlapping and distinct substrate specificities J Lipid Res 2005 5 1239 1247 10.1194/jlr.M400511-JLR200 15772423

[B47] Gouedard C Koum-Besson N Barouki R Morel Y Opposite regulation of the human paraoxonase-1 gene PON-1 by fenofibrate and statins Mol Pharmacol 2003 5 945 956 10.1124/mol.63.4.945 12644596

[B48] Khersonsky O Tawfik DS Structure-reactivity studies of serum paraoxonase PON1 suggest that its native activity is lactonase Biochemistry 2005 5 6371 6382 10.1021/bi047440d 15835926

[B49] Christians U Jacobsen W Floren LC Metabolism and drug interactions of 3-hydroxy-3-methylglutaryl coenzyme A reductase inhibitors in transplant patients: are the statins mechanistically similar? Pharmacol Ther 1998 5 1 34 10.1016/S0163-7258(98)00016-3 9804052

[B50] Jacobsen W Kuhn B Soldner A Kirchner G Sewing KF Kollman PA Benet LZ Christians U Lactonization is the critical first step in the disposition of the 3-hydroxy-3-methylglutaryl-CoA reductase inhibitor atorvastatin Drug Metab Dispos 2000 5 1369 1378 11038166

[B51] Fujino H Saito T Tsunenari Y Kojima J Sakaeda T Metabolic properties of the acid and lactone forms of HMG-CoA reductase inhibitors Xenobiotica 2004 5 961 971 10.1080/00498250400015319 15801541

[B52] Park JE Kim KB Bae SK Moon BS Liu KH Shin JG Contribution of cytochrome P450 3A4 and 3A5 to the metabolism of atorvastatin Xenobiotica 2008 5 1240 1251 10.1080/00498250802334391 18720283

[B53] Grube M Kock K Oswald S Draber K Meissner K Eckel L Bohm M Felix SB Vogelgesang S Jedlitschky G Siegmund W Warzok R Kroemer HK Organic anion transporting polypeptide 2B1 is a high-affinity transporter for atorvastatin and is expressed in the human heart Clinical Pharmacology & Therapeutics 2006 5 607 620 10.1016/j.clpt.2006.09.010 17178262

[B54] Lau YY Huang Y Frassetto L Benet LZ effect of OATP1B transporter inhibition on the pharmacokinetics of atorvastatin in healthy volunteers Clin Pharmacol Ther 2007 5 194 204 10.1038/sj.clpt.6100038 17192770

[B55] Nishimura M Naito S Tissue-specific mRNA expression profiles of human ATP-binding cassette and solute carrier transporter superfamilies Drug Metab Pharmacokinet 2005 5 452 477 10.2133/dmpk.20.452 16415531

[B56] Knauer MJ Urquhart BL Meyer zu Schwabedissen HE Schwarz UI Lemke CJ Leake BF Kim RB Tirona RG Human skeletal muscle drug transporters determine local exposure and toxicity of statins Circ Res 2010 5 297 306 10.1161/CIRCRESAHA.109.203596 19940267

[B57] Hilgendorf C Ahlin G Seithel A Artursson P Ungell AL Karlsson J Expression of thirty-six drug transporter genes in human intestine, liver, kidney, and organotypic cell lines Drug Metabolism and Disposition 2007 5 1333 1340 10.1124/dmd.107.014902 17496207

[B58] Li L Lee TK Meier PJ Ballatori N Identification of glutathione as a driving force and leukotriene C4 as a substrate for oatp1, the hepatic sinusoidal organic solute transporter J Biol Chem 1998 5 16184 16191 10.1074/jbc.273.26.16184 9632674

[B59] Mahagita C Grassl SM Piyachaturawat P Ballatori N Human organic anion transporter 1B1 and 1B3 function as bidirectional carriers and do not mediate GSH-bile acid cotransport Am J Physiol Gastrointest Liver Physiol 2007 5 G271 278 10.1152/ajpgi.00075.2007 17412826

[B60] Kilford PJ Gertz M Houston JB Galetin A Hepatocellular binding of drugs: Correction for unbound fraction in hepatocyte incubations using microsomal binding or drug lipophilicity data Drug Metabolism and Disposition 2008 5 1194 1197 10.1124/dmd.108.020834 18411401

[B61] Bogman K Peyer AK Torok M Kusters E Drewe J HMG-CoA reductase inhibitors and P-glycoprotein modulation Br J Pharmacol 2001 5 1183 1192 10.1038/sj.bjp.0703920 11250868 PMC1572659

[B62] Boyd RA Stern RH Stewart BH Wu X Reyner EL Zegarac EA Randinitis EJ Whitfield L Atorvastatin coadministration may increase digoxin concentrations by inhibition of intestinal P-glycoprotein-mediated secretion J Clin Pharmacol 2000 5 91 98 10.1177/00912700022008612 10631627

[B63] Chen C Mireles RJ Campbell SD Lin J Mills JB Xu JJ Smolarek TA Differential interaction of 3-hydroxy-3-methylglutaryl-coa reductase inhibitors with ABCB1, ABCC2, and OATP1B1 Drug Metab Dispos 2005 5 537 546 10.1124/dmd.104.002477 15616150

[B64] Hochman JH Pudvah N Qiu J Yamazaki M Tang C Lin JH Prueksaritanont T Interactions of human P-glycoprotein with simvastatin, simvastatin acid, and atorvastatin Pharm Res 2004 5 1686 1691 15497697 10.1023/b:pham.0000041466.84653.8c

[B65] Sakaeda T Fujino H Komoto C Kakumoto M Jin JS Iwaki K Nishiguchi K Nakamura T Okamura N Okumura K Effects of acid and lactone forms of eight HMG-CoA reductase inhibitors on CYP-mediated metabolism and MDR1-mediated transport Pharmaceutical Research 2006 5 506 512 10.1007/s11095-005-9371-5 16388406

[B66] Chandra P Brouwer KLR The complexities of hepatic drug transport: Current knowledge and emerging concepts Pharmaceutical Research 2004 5 719 735 15180326 10.1023/b:pham.0000026420.79421.8f

[B67] Hamelin BA Turgeon J Hydrophilicity/lipophilicity: relevance for the pharmacology and clinical effects of HMG-CoA reductase inhibitors Trends in Pharmacological Sciences 1998 5 26 37 10.1016/S0165-6147(97)01147-4 9509899

[B68] Austin RP Barton P Mohmed S Riley RJ The binding of drugs to hepatocytes and its relationship to physicochemical properties Drug Metab Dispos 2005 5 419 425 15616151 10.1124/dmd.104.002436

[B69] Hallifax D Houston JB Uptake and intracellular binding of lipophilic amine drugs by isolated rat hepatocytes and implications for prediction of in vivo metabolic clearance Drug Metab Dispos 2006 5 1829 1836 10.1124/dmd.106.010413 16882765

[B70] Schwartlander R Schmid J Brandenburg B Katenz E Wolfgang F Vondran R Pless G Cheng XD Pascher A Neuhaus P Sauer IM Continuously microscopically observed and process-controlled cell culture within the SlideReactor: Proof of a new concept for cell characterization Tissue Engineering 2007 5 187 196 10.1089/ten.2006.0071 17518592

[B71] Toutain PL Bousquet-Melou A Free drug fraction vs free drug concentration: a matter of frequent confusion J Vet Pharmacol Ther 2002 5 460 463 10.1046/j.1365-2885.2002.00442.x 12485352

[B72] Bartholome K Rius M Letschert K Keller D Timmer J Keppler D Data-based mathematical modeling of vectorial transport across double-transfected polarized cells Drug Metab Dispos 2007 5 1476 1481 10.1124/dmd.107.015636 17548463

[B73] Poland J Zell A Main Vector Adaptation: A CMA Variant with Linear Time and Space Complexity Proceedings of the Genetic and Evolutionary Computation Conference (GECCO-2001); Washington, D.C., USA 2001 312 317

[B74] Deuflhard P Hairer E Zugck J One-Step and Extrapolation Methods for Differential-Algebraic Systems Numerische Mathematik 1987 5 501 516 10.1007/BF01400352

[B75] Prueksaritanont T Ma B Fang X Subramanian R Yu J Lin JH beta-Oxidation of simvastatin in mouse liver preparations Drug Metab Dispos 2001 5 1251 1255 11560866

[B76] Deng WJ Nie S Dai J Wu JR Zeng R Proteome, phosphoproteome, and hydroxyproteome of liver mitochondria in diabetic rats at early pathogenic stages Mol Cell Proteomics 2010 5 100 116 10.1074/mcp.M900020-MCP200 19700791 PMC2808256

[B77] Motawi TM Hashem RM Rashed LA El-Razek SM Comparative study between the effect of the peroxisome proliferator activated receptor-alpha ligands fenofibrate and n-3 polyunsaturated fatty acids on activation of 5'-AMP-activated protein kinase-alpha1 in high-fat fed rats J Pharm Pharmacol 2009 5 1339 1346 19814866 10.1211/jpp/61.10.0010

[B78] Bradford MM A rapid and sensitive method for the quantitation of microgram quantities of protein utilizing the principle of protein-dye binding Anal Biochem 1976 5 248 254 10.1016/0003-2697(76)90527-3 942051

[B79] Lins RL Matthys KE Verpooten GA Peeters PC Dratwa M Stolear JC Lameire NH Pharmacokinetics of atorvastatin and its metabolites after single and multiple dosing in hypercholesterolaemic haemodialysis patients Nephrol Dial Transplant 2003 5 967 976 10.1093/ndt/gfg048 12686673

[B80] Launay-Vacher V Izzedine H Deray G Statins' dosage in patients with renal failure and cyclosporine drug-drug interactions in transplant recipient patients Int J Cardiol 2005 5 9 17 10.1016/j.ijcard.2004.04.005 15860377

[B81] Kantola T Kivisto KT Neuvonen PJ Effect of itraconazole on the pharmacokinetics of atorvastatin Clin Pharmacol Ther 1998 5 58 65 10.1016/S0009-9236(98)90023-6 9695720

[B82] Tirona RG Leake BF Merino G Kim RB Polymorphisms in OATP-C: identification of multiple allelic variants associated with altered transport activity among European- and African-Americans J Biol Chem 2001 5 35669 35675 10.1074/jbc.M103792200 11477075

[B83] Cui YH Konig J Keppler D Vectorial transport by double-transfected cells expressing the human uptake transporter SLC21A8 and the apical export pump ABCC2 Molecular Pharmacology 2001 5 934 943 11641421 10.1124/mol.60.5.934

