# Supplementary material for: A systems biology approach to dynamic modeling and inter-subject variability of statin pharmacokinetics in human hepatocytes
Source: BMC Syst Biol. 2011 May 6;5:66. doi: 10.1186/1752-0509-5-66 (PMC3117731; doi:10.1186/1752-0509-5-66)
Supplement: Additional file 1 — Derivation of atorvastatin kinetics at CYP3A4. This file contains the derivation of the atorvastatin kinetics at CYP3A4, which describes the competition of alternative substrate degradation to alternative products (supplemented as .pdf-file). [file 1752-0509-5-66-S1.PDF]

# Additional file 1 - Derivation of Atorvastatin kinetics at CYP3A4

Atorvastatin acid (AS) and lactone (ASL) are hydroxylated to the corresponding para- and ortho-hydroxy-metabolites (ASpOH and ASoOH, ASLpOH and ASLoOH), mediated by CYP3A4. Since both AS and ASL are both detoxified by the same enzyme, CYP3A4, a kinetics describing the alternative conversion of both substrates has to be considered. Furthermore, since both AS and ASL are detoxified to two metabolites, respectively, it can not only be characterized as an alternative substrate conversion, but also as an alternative substrate conversion to alternative products. The enzymatic reaction scheme is depicted in **Figure 1**.

The mass balance equation for CYP3A4 enzyme ("E") is formulated as

$$c_{E,t} = c_E + c_{(ASE)_1} + c_{(ASE)_2} + c_{(ASLE)_1} + c_{(ASLE)_2} \quad , \qquad (1)$$

where the total amount of enzyme E is the sum of free enzyme amount $c_E$ and the different enzyme-substrate complexes.

The acid metabolite ASpOH is formed by one reaction with the rate

$$r_{3A4,ASpOH} = k_{+2,AS,1} \cdot c_{(ASE)_1} \quad . \qquad (2)$$

Dividing left-hand side and right-hand side of equation (2) by equation (1) we get

$$\frac{r_{3A4,ASpOH}}{c_{E,t}} = \frac{k_{+2,AS,1} \cdot c_{(ASE)_1}}{c_E + c_{(ASE)_1} + c_{(ASE)_2} + c_{(ASLE)_1} + c_{(ASLE)_2}} \qquad (3)$$

The enzyme-substrate-complexes can be broken down by introduction of the Michaelis-Menten-constant of the substrate AS-enzyme complex $(ASE)_1$,

$$K_{M,3A4,ASpOH} = \frac{k_{-1,AS,1} + k_{+2,AS,1}}{k_{+1,AS,1}} = \frac{c_{AS} \cdot c_E}{c_{(ASE)_1}} \quad , \qquad (4)$$

of the Michaelis-Menten-constant of the substrate AS-enzyme complex $(ASE)_2$,

$$K_{M,3A4,ASoOH} = \frac{k_{-1,AS,2} + k_{+2,AS,2}}{k_{+1,AS,2}} = \frac{c_{AS} \cdot c_E}{c_{(ASE)_2}} \quad , \qquad (5)$$

of the Michaelis-Menten-constant of the ASL-enzyme complex $(ASLE)_1$,

$$K_{M,3A4,ASLpOH} = \frac{k_{-1,ASL,1} + k_{+2,ASL,1}}{k_{+1,ASL,1}} = \frac{c_{ASL} \cdot c_E}{c_{(ASLE)_1}} \quad , \qquad (6)$$

and of the Michaelis-Menten-constant of the ASL-enzyme complex $(ASLE)_{2,}$

$$K_{M,3A4,ASLoOH} = \frac{k_{-1,ASL,2} + k_{+2,ASL,2}}{k_{+1,ASL,2}} = \frac{c_{ASL} \cdot c_E}{c_{(ASLE)_2}} \ . \tag{7}$$

Applying equations (4), (5), (6) and (7) to equation (3) it follows

$$\frac{r_{3A4,ASpOH}}{c_{E,t}} = \frac{k_{+2,AS,1} \cdot \frac{c_{AS} \cdot c_E}{K_{M,3A4,ASpOH}}}{c_E + \frac{c_{AS} \cdot c_E}{K_{M,3A4,ASpOH}} + \frac{c_{AS} \cdot c_E}{K_{M,3A4,ASoOH}} + \frac{c_{ASL} \cdot c_E}{K_{M,3A4,ASLpOH}} + \frac{c_{ASL} \cdot c_E}{K_{M,3A4,ASLoOH}}} \ . \tag{8}$$

The maximal rate of ASpOH formation is reached, when the whole amount of enzyme E is used for the formation of the AS-enzyme-complex, $(ASE)_1$, formulated as

$$r_{\max,3A4,ASpOH} = k_{+2,AS,1} \cdot c_{E,t} \ , \tag{9}$$

which can be applied to equation (A.8), leading to

$$r_{3A4,ASpOH} = \frac{r_{\max,3A4,ASpOH} \cdot \frac{c_{AS}}{K_{M,3A4,ASpOH}}}{1 + \frac{c_{AS}}{K_{M,3A4,ASpOH}} + \frac{c_{AS}}{K_{M,3A4,ASoOH}} + \frac{c_{ASL}}{K_{M,3A4,ASLpOH}} + \frac{c_{ASL}}{K_{M,3A4,ASLoOH}}} \ , \tag{A.10}$$

which finally describes the reaction kinetics of the hydroxylation of AS to ASpOH with the dominator

$$D = 1 + \frac{c_{AS}}{K_{M,3A4,ASpOH}} + \frac{c_{AS}}{K_{M,3A4,ASoOH}} + \frac{c_{ASL}}{K_{M,3A4,ASLpOH}} + \frac{c_{ASL}}{K_{M,3A4,ASLoOH}} \ . \tag{11}$$

Similarly, the reaction kinetics of formation of ASoOH, of ASLpOH, and of ASLoOH can be formulated as

$$r_{3A4,ASoOH} = \frac{r_{\max,3A4,ASoOH} \cdot \frac{c_{AS}}{K_{M,3A4,ASoOH}}}{D} \ , \tag{12}$$

$$r_{3A4,ASLpOH} = \frac{r_{\max,3A4,ASLpOH} \cdot \frac{c_{AS}}{K_{M,3A4,ASLpOH}}}{D} \ \text{and} \tag{13}$$

$$r_{3A4,ASLoOH} = \frac{r_{\max,3A4,ASoOH} \cdot \frac{c_{AS}}{K_{M,3A4,ASLoOH}}}{D} \ . \tag{14}$$

Thus, the rate of degradation of the substrate AS at CYP3A4 can be formulated as

$$r_{3A4,AS} = -\frac{r_{\max,3A4,ASpOH} \cdot \frac{c_{AS}}{K_{M,3A4,ASpOH}}}{D} - \frac{r_{\max,3A4,ASoOH} \cdot \frac{c_{AS}}{K_{M,3A4,ASoOH}}}{D} \ , \tag{15}$$

and the rate of degradation of the substrate ASL at CYP3A4 can be formulated as

$$r_{3A4,ASL} = -\frac{r_{\max,3A4,ASLpOH} \cdot \dfrac{c_{AS}}{K_{M,3A4,ASpOH}}}{D} - \frac{r_{\max,3A4,ASLoOH} \cdot \dfrac{c_{AS}}{K_{M,3A4,ASoOH}}}{D} \quad . \tag{16}$$

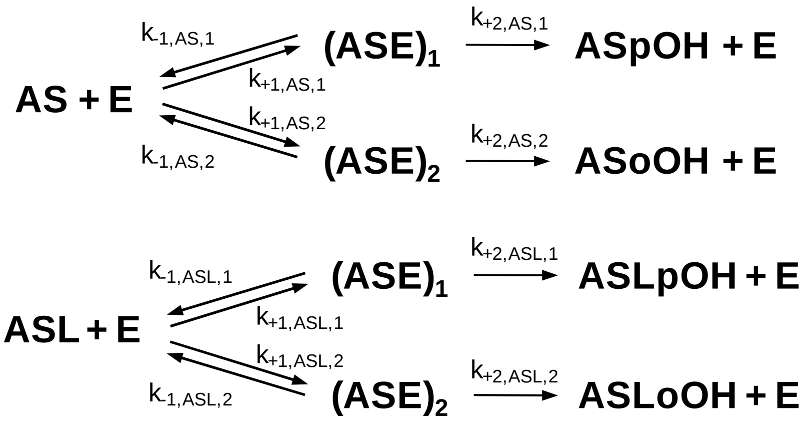


**Figure 1 - Reaction scheme of AS and ASL hydroxylation at CYP3A4**

AS and ASL, are hydroxylated to the corresponding para- and ortho-hydroxy-metabolites, ASpOH and ASoOH, ASLpOH and ASLoOH mediated by CYP3A4 ("E"). Since hydroxylation takes place at two different positions, para- and ortho-, the enzyme-substrate-complexes are different between para- (index 1) and ortho- (index 2) hydroxyl-metabolites. We assume, that the dissociation of the enzyme-substrate-complex is much faster than the formation of the hydroxy-metabolite, respectively. Hereby, substrate, enzyme, and enzyme-substrate-complex are assumed to be in equilibrium, respectively. Rate constants of each reaction are used in the derivation of the kinetics as indicated in the figure.
