# Supplementary material for: A systems biology approach to dynamic modeling and inter-subject variability of statin pharmacokinetics in human hepatocytes
Source: BMC Syst Biol. 2011 May 6;5:66. doi: 10.1186/1752-0509-5-66 (PMC3117731; doi:10.1186/1752-0509-5-66)
Supplement: Additional file 2 — Derivation of kinetics of unspecific binding to macromolecules. This file contains the derivation of kinetics of the unspecific binding of atorvastatin metabolites to macromolecules, for example proteins (supplemented as .pdf-file). [file 1752-0509-5-66-S2.PDF]

Additional file 2 - Derivation of kinetics of unspecific protein binding

Atorvastatin acid (AS) and lactone (ASL) and the corresponding para- and ortho-hydroxy-metabolites (ASpOH and ASoOH, ASLpOH and ASLoOH) are subject to unspecific binding to macromolecules, like proteins. The association to macromolecules (*as*) and dissociation from the macromolecules (*dis*) can be formulated as

$$r_{bind,j} = k_{as} \cdot c_{Ma} \cdot c_j^c - k_{dis} \cdot c_{Ma,j} \quad . \qquad (1)$$

with the macromolecule concentration c_{Ma}, the free, unbound, concentration c_j^c, and the macromolecule-drug-complex concentration $c_{Ma,j}$ inside the hepatocytes. Substituting the macromolecule-drug-complex concentration $c_{Ma,j}$ with the bound concentration of drug, c_j^b, and assuming, that the macromolecule concentration inside the cell is constant, equation (1) can be formulated as

$$r_{bind,j} = k_{as} \cdot c_j^c - k_{dis} \cdot c_j^b \quad . \qquad (2)$$

We further assume, that the association and dissociation of the unspecific binding are very fast compared to reaction and transport kinetics, and therefore are in equilibrium (eq) with the equilibrium constant

$$K_B = \frac{k_{dis}}{k_{as}} = \frac{c_{j,eq}^c}{c_{j,eq}^b} \quad . \qquad (3)$$

Introducing the fraction unbound fu_j of the metabolite j

$$fu_j = \frac{c_{j,eq}^c}{c_{j,eq}^c + c_{j,eq}^b} \quad , \qquad (4)$$

which describes the relation of intracellular free to sum of free and bound concentration in equilibrium, equation (3) can be written as

$$\frac{k_{dis}}{k_{as}} = \frac{c_{j,eq}^c}{c_{j,eq}^b} = \frac{fu_j}{1 - fu_j} \quad . \qquad (5)$$

Substituting k_{as} in equation (2) with rearranged equation (5) as

$$k_{as} = k_{dis} \frac{1 - fu_j}{fu_j} \quad , \qquad (6)$$

results in

$$r_{bind,j} = k_{dis} \frac{1 - fu_j}{fu_j} \cdot c_j^c - k_{dis} \cdot c_j^b = k_{dis} \cdot \left(\left(\frac{1}{fu_j} - 1 \right) \cdot c_j^c - c_j^b \right), \quad (7)$$

which describes the kinetics for the unspecific binding of Atorvastatin and metabolites inside the hepatocytes used in the modeling. The kinetics is constrained by $0 < fu_j < 1$, where a value nearer to 0 indicate a higher binding affinity and a value nearer to 1 a lower binding affinity of compound *j*.
