# Supplementary material for: A systems biology approach to dynamic modeling and inter-subject variability of statin pharmacokinetics in human hepatocytes
Source: BMC Syst Biol. 2011 May 6;5:66. doi: 10.1186/1752-0509-5-66 (PMC3117731; doi:10.1186/1752-0509-5-66)
Supplement: Additional file 3 — Atorvastatin metabolite concentrations from the time-series experiment on primary human hepatocytes of individual 1. Extracellular concentrations (upper part) and intracellular concentrations (lower part) of atorvastatin acid and lactone (AS and ASL) and corresponding para- and ortho-hydroxy-metabolites (acids: ASpOH and ASoOH; lactones: ASLpOH and ASLoOH) at the defined time-points with mean and standard deviation (n = 3) from triplicate measurements per LC-MS/MS (n.d.: not determinable; n.o.: not observed). The recovery is calculated from material balance equations and is defined as the sum of intracellular and extracellular metabolite amounts at the respective time-point divided by initial AS amount (supplemented as .pdf-file). [file 1752-0509-5-66-S3.PDF]

Supplemental table S1: Atorvastatin metabolite concentrations in the time-series experiment on primary human hepatocytes of Individual 1

	AS		ASL		ASpOH		ASoOH		ASLpOH		ASLoOH		
time [min]	Extracellular Concentrations [pmol ml^{-1}]												Recovery [%]
0	8797.1		30.5		n.d.		n.d.		n.d.		n.d.		
10	8258.3	±405.1	28.5	±4.9	n.d.		3.3	±0.2	n.d.		n.d.		97.6
30	8086.6	±518.3	31.3	±5.3	11.0	±0.5	41.0	±0.9	n.d.		n.d.		96.8
60	7648.4	±1473.8	41.4	±2.6	29.8	±3.3	167.4	±17.5	5.2	±0.1	9.0	±0.2	94.8
120	6132.4	±171.2	54.1	±1.8	241.6	±7.5	899.9	±19.1	10.1	±0.5	21.5	±0.4	88.8
180	5592.2	±254.3	61.7	±0.6	337.1	±19.7	1205.0	±37.8	11.4	±0.8	24.1	±1.9	86.5
240	5190.3	±189.3	62.0	±4.6	516.2	±11.9	1675.5	±63.5	13.7	±0.6	25.5	±0.9	89.0
300	4404.2	±230.1	55.9	±4.4	723.4	±30.2	2201.0	±66.3	16.2	±1.0	29.3	±0.4	89.0
360	3285.1	±352.2	51.3	±7.3	868.4	±61.0	2523.1	±76.3	15.4	±0.9	36.3	±2.4	80.8
480	n.o.		n.o.		n.o.		n.o.		n.o.		n.o.		
600	2073.0	±184.9	42.0	±4.9	1245.7	±38.2	3146.5	±91.1	21.2	±1.4	45.8	±6.2	78.2
time [min]	Intracellular Concentrations [pmol ml^{-1}]												
10	39727.4	±2185.2	418.6	±9.7	379.7	±18.4	516.1	±78.1	194.1	±11.1	n.d.	n.d.	
30	41668.0	±2317.2	566.8	±72.0	2740.9	±167.4	3029.1	±186.8	237.9	±18.7	n.d.	n.d.	
60	49772.4	±1749.1	709.0	±23.5	4737.3	±233.1	6526.6	±657.6	356.6	±38.4	213.7	±49.7	
120	44509.8	±2992.8	766.6	±25.4	8344.8	±177.9	9949.9	±834.8	527.6	±34.1	340.5	±39.4	
180	34790.4	±3072.2	780.7	±83.4	7872.6	±964.4	8711.4	±1062.5	502.5	±35.1	339.5	±6.2	
240	29716.1	±3144.4	757.2	±34.4	8576.3	±1190.3	8411.1	±861.5	531.8	±34.1	385.8	±35.4	
300	35702.4	±1663.2	654.0	±71.6	9728.0	±931.4	8887.3	±809.0	588.1	±47.4	280.8	±115.7	
360	27020.0	±909.3	625.0	±142.6	9767.9	±439.4	8665.5	±394.5	529.4	±53.4	286.6	±26.3	
480	25491.5	±1835.9	622.7	±13.9	13298.5	±309.4	11235.2	±787.7	596.5	±25.7	442.7	±53.8	
600	18839.0	±1056.5	520.2	±60.1	13176.6	±1601.2	9775.0	±987.2	539.7	±38.1	410.3	±54.1	

Extracellular concentrations (upper part) and intracellular concentrations (lower part) of Atorvastatin metabolites, Atorvastatin acid and lactone (AS and ASL) and corresponding para- and ortho-hydroxy-metabolites (acids: ASpOH and ASoOH; lactones: ASLpOH and ASLoOH) at the defined time-points with mean and standard deviation (n=3) from measurements per LC-MS/MS (n.d.: not determinable; n.o.: not observed). The recovery calculated from material balance equations is defined as the sum of intracellular and extracellular metabolite amounts divided by initial AS amount.
