# Supplementary material for: A systems biology approach to dynamic modeling and inter-subject variability of statin pharmacokinetics in human hepatocytes
Source: BMC Syst Biol. 2011 May 6;5:66. doi: 10.1186/1752-0509-5-66 (PMC3117731; doi:10.1186/1752-0509-5-66)
Supplement: Additional file 4 — Analysis of parameter sensitivity and following model reduction procedure. This file contains the analysis of parameter sensitivity and the exemplary model reduction procedure, which is necessary for achieving a high quality predictive model of atorvastatin metabolism (supplemented as .pdf-file). [file 1752-0509-5-66-S4.PDF]

Additional file 4 - Analysis of parameter sensitivity and following model reduction procedure

Methods

Parameter sensitivity and identifiability analysis

The local identifiability of the optimized model parameters was estimated using the Fisher-Information-Matrix (**FIM**) [1-7]

$$\mathbf{FIM} = \sum_{n=1}^{N} \left(\frac{\partial \mathbf{y}(t_n, \mathbf{p})}{\partial \mathbf{p}} \right)^T \mathbf{Q}(t_n) \left(\frac{\partial \mathbf{y}(t_n, \mathbf{p})}{\partial \mathbf{p}} \right) , \tag{1}$$

which is composed of the sensitivity matrix

$$\mathbf{S}_n = \frac{\partial \mathbf{y}(t_n, \mathbf{p})}{\partial \mathbf{p}} \tag{2}$$

and the weighting matrix $\mathbf{Q}(t_n)$, at the experimental time points t_n, respectively. The sensitivity matrix $\mathbf{S}_n$ comprises the first-order derivatives of the state variables (concentrations) at the defined time points, with respect to the model parameters. The weighting matrix $\mathbf{Q}(t_n)$ represents the inverse of the measurement error covariance matrix.

Based on the **FIM**, the so called Top-Down-Classification [8] can be performed, identifying parameters, which cause high estimation errors. To this end, **FIM** has to be normalized with the diagonal matrix of the nominal parameters, resulting in the normalized Fisher-Information-Matrix ($\mathbf{FIM}^*$) [5]:

$$\mathbf{FIM}^* = diag(\mathbf{p})^T \cdot \mathbf{FIM} \cdot diag(\mathbf{p}) \ . \tag{3}$$

Since **FIM** presents also the inverse of the parameter estimation error covariance matrix of the best linear unbiased estimator (BLUE) [6]

$$\mathbf{C} = \mathbf{FIM}^{-1} \ , \tag{4}$$

the inverse of the normalized Fisher-Information-Matrix $\mathbf{FIM}^*$ (equation (3)) can be defined as normalized estimation error covariance matrix $\mathbf{C}^*$

$$\mathbf{C}^* = (\mathbf{FIM}^*)^{-1} , \tag{5}$$

which gives a lower bound for the relative parameter errors ε_{p_l} [9],

$$\varepsilon_{p_l}^2 \geq ((\mathbf{FIM}^*)^{-1})_{ll} , \tag{6}$$

due to the Cramer-Rao-inequality [3, 4, 7].

Further, information on parameter correlations can be extracted from the correlation matrix

$$R_{kl} = \frac{C_{kl}^*}{\sqrt{C_{kk}^* \cdot C_{ll}^*}} , \qquad R_{kl} = 1; k = l \tag{7}$$

which comprises the correlation coefficients between the *k*th and the *l*th parameters [6]. The correlation matrix gives information about highly correlated parameters, of which variations in one parameter value, leading to a change in the state variables, can be compensated by the correlating partner.

Further, the reciprocal condition number of the **FIM*** (equation (3)),

$$rcond(\mathbf{FIM}^*) = \frac{\lambda_{\min}}{\lambda_{\max}} , \tag{8}$$

which is defined by the ratio between the minimal and the maximal eigenvalue of the **FIM***, indicates if the **FIM*** is conditioned well. If the **FIM*** is well conditioned, respectively identifiable, the reciprocal condition number *rcond*(**FIM***) is near 1 and if the **FIM*** is badly conditioned, respectively non-identifiable, *rcond*(**FIM***) is near to zero [6, 7].

Additionally to the **FIM** estimation, normalized sensitivity coefficients

$$S_{j,l,n}^{norm} = \frac{\partial y_j(t_n)}{\partial p_l} \frac{p_l}{y_j} \tag{9}$$

are calculated at equidistant distributed time points in the experimental time-frame and the absolute values are summed up over all time-points n and all state variables j to gain the normalized parameter sensitivities:

$$S_l^{norm} = \sum_j^J \sum_n^N \left| S_{j,l,n}^{norm} \right| . \tag{10}$$

The Fisher-Information-Matrix (equation (3)) as well as the normalized parameter sensitivities (equation (10)) were calculated in FORTRAN and transferred to MATLAB for the matrix operations described above. The reciprocal condition number (equation (8)) is calculated in MATLAB with the LAPACK-function "rcond".

In this study the sensitivities were not calculated analytically, but approximated by a centered difference quotient

$$S_{j,l} = \frac{\partial y_j(t_n)}{\partial p_l} \approx \frac{y_j(p+\Delta p) - y_j(p-\Delta p)}{2 \cdot \Delta p_l} , \tag{11}$$

where the change in the state variable is caused by a small perturbation Δp_l of the parameter p_l.

Results

Parameter sensitivity and local identifiability

While the model provides a concise representation of the major and experimentally confirmed reactions, it may suffer from two fundamental parameter estimation problems: sensitivity and correlation between parameters. Both need to be studied carefully to provide evidence that the parameters are adequately identifiable from the data and thus strengthen the credibility of the model. Despite its importance, parameter identifiability has been rarely addressed in the literature of pharmacokinetic modeling. We believe that this issue needs to be tackled in appropriate depth to create

a framework of consistency of the model, essential for more precise predictions in toxicology.

To get a quantitative measure of the parameter quality, criteria based on the normalized Fisher-Information-Matrix (**FIM***) (equation (3)) and normalized parameter sensitivities (equation (10)) were calculated. The **FIM*** represents the information content of the underlying experimental data, and derived criteria, like the relative parameter errors (equation (6)), the parameter correlation matrix (equation (7)), and the condition number of the **FIM*** (equation (8)), as well as the Top-Down-Classification [8], describe the local identifiability of the parameters.

The normalized parameter sensitivities in equation (10) and the **FIM*** parameter sensitivities in equation (2) were approximated by the difference quotient in equation (11) with respect to a small perturbation factor Δp_l of each parameter p_l of 0.1%, except the a-priori identified parameters (**Table A**) which are not considered in the local sensitivity analysis.

Table A - Parameters which were fixed in the optimization procedure to literature values

Parameter	Value	Units	Literature
$K_{M,3A4,ASpOH}$	25600	pmol·ml^{-1}	[10]
$K_{M,3A4,ASoOH}$	29700	pmol·ml^{-1}	[10]
$K_{M,3A4,ASLpOH}$	1400	pmol·ml^{-1}	[10]
$K_{M,3A4,ASLoOH}$	3900	pmol·ml^{-1}	[10]
$K_{M,1B1,AS}$	18900	pmol·ml^{-1}	[11]
$K_{M,2B1,AS}$	200	pmol·ml^{-1}	[12]
$K_{M,1A3,AS}$	12000	pmol·ml^{-1}	[13]
$K_{I,1A3,AS}$	75000	pmol·ml^{-1}	[13]
k_{CR}	0.0025	min^{-1}	[14]
k_{dis}	600	min^{-1}	[15]

The normalized parameter sensitivities (**Figure A**) demonstrate, that the maximal rate coefficient $r_{max,1A3,AS}$ of UGT1A3 mediated lactonization has the highest influence on the biotransformation network with a normalized sensitivity of 1090.0, and that the

maximal rate coefficient of the OATP2B1 mediated import of AS, $r_{max,2B1,AS}$, has the lowest influence on the biotransformation network with a sensitivity of $4.8 \cdot 10^{-3}$. Totally, three of the parameters, $r_{max,ex,AS}$, $r_{max,2B1,AS}$, $K_{M,ex,AS}$, have very low local sensitivities. The other parameters have a high influence on the biotransformation metabolism. However, examining the sensitivities of the individual enzymes, the parameters of the CYP3A4-hydroxylation and UGT1A3-lactonization have higher normalized sensitivities, averaged 648.4, than the parameters of active transport and passive diffusion, averaged 130.7.

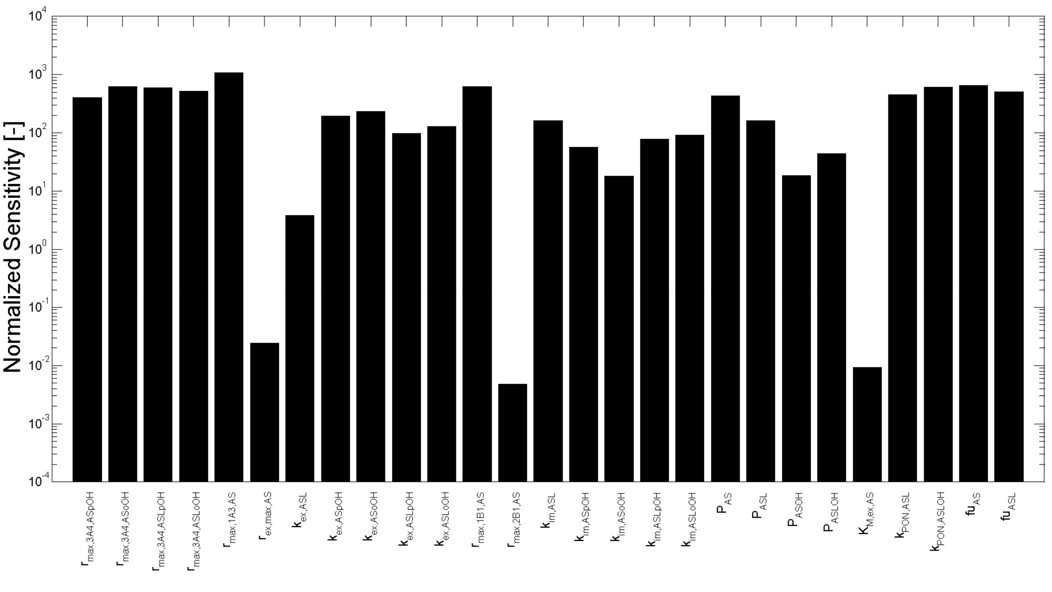

Figure A - Normalized parameter sensitivities of the full model of atorvastatin metabolism

The normalized parameter sensitivities are of the full model of atorvastatin metabolism, comprising both estimated and fixed parameters as obtained in the optimization procedure. High values indicate a high sensitivity of the respective parameter, whereas low values indicate, that the respective parameter is insensitive.

The correlation matrix (**Figure B**), comprising the correlation coefficients between the parameters, displays that the parameters are highly correlated with 54 correlation coefficients being higher than 0.7. Even more, the correlation matrix presents 23

entries with values higher than 0.99, indicating once more that there are severe identifiability difficulties.

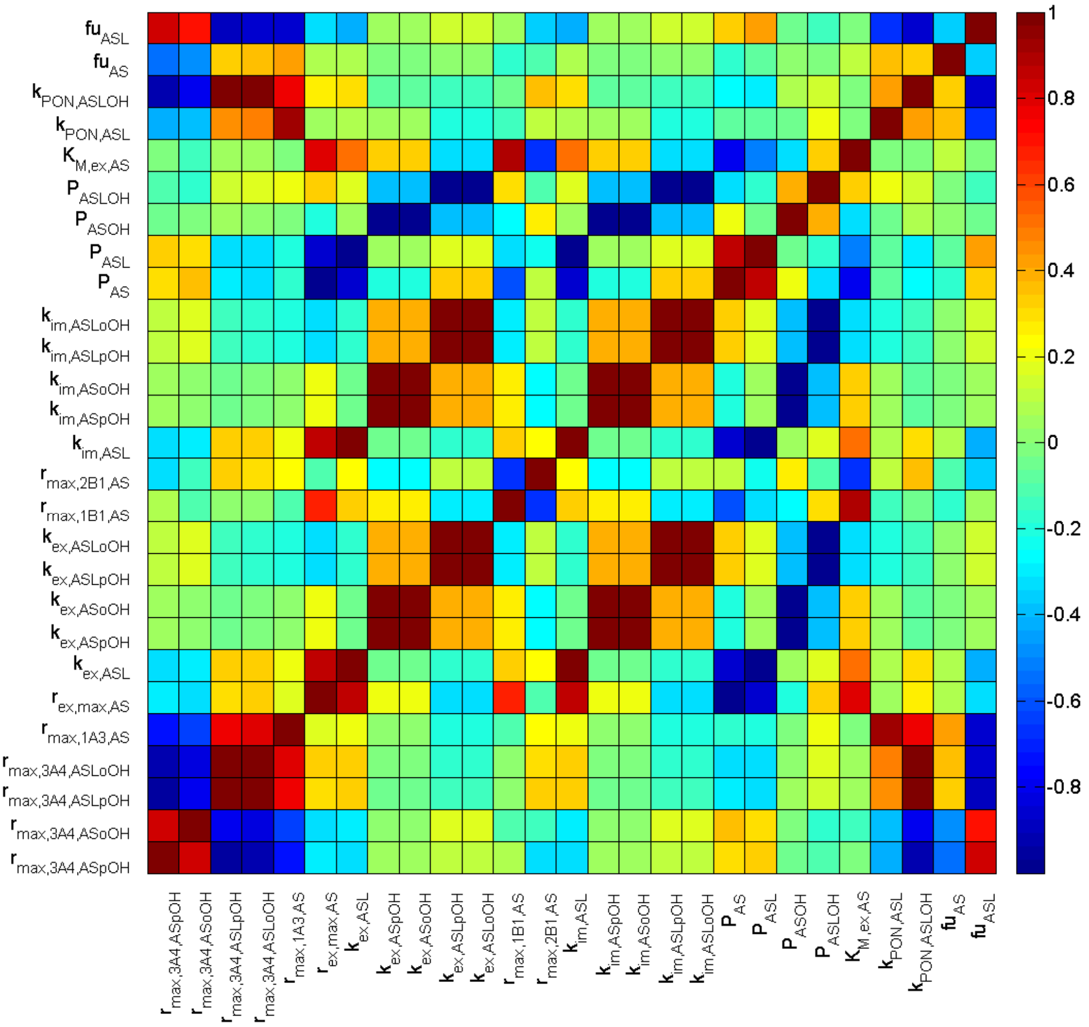

Figure B - Correlation matrix of the of the full model of Atorvastatin metabolism

The correlation matrix is displayed as pseudo-color plot of the parameters of the full model against themselves and comprises the single correlation coefficients between two parameters, respectively. Dark blue and dark red colors display parameters with high negative and positive linear dependencies, respectively.

The relative parameter errors are estimated from the inverse of the **FIM*** (equation (6)). In this case of the full model, the relative parameter errors are very high, averaged $1.8 \cdot 10^6$ % over all parameters with a bandwidth from minimal 3 % to maximal $1.17 \cdot 10^7$ % (**Figure C**). Only 11 of 37 parameters have relative errors under

100%, which are the parameters of reaction, protein binding and the maximal rate coefficient of the OATP2B1 mediated import of AS, $r_{max,2B1,AS}$. Consequently, Top-down classification, analysis of the correlation matrix and analysis of relative parameter errors show that the verified full model is hardly to identify, which is obviously caused by highly correlated parameters in the transport steps of the system.

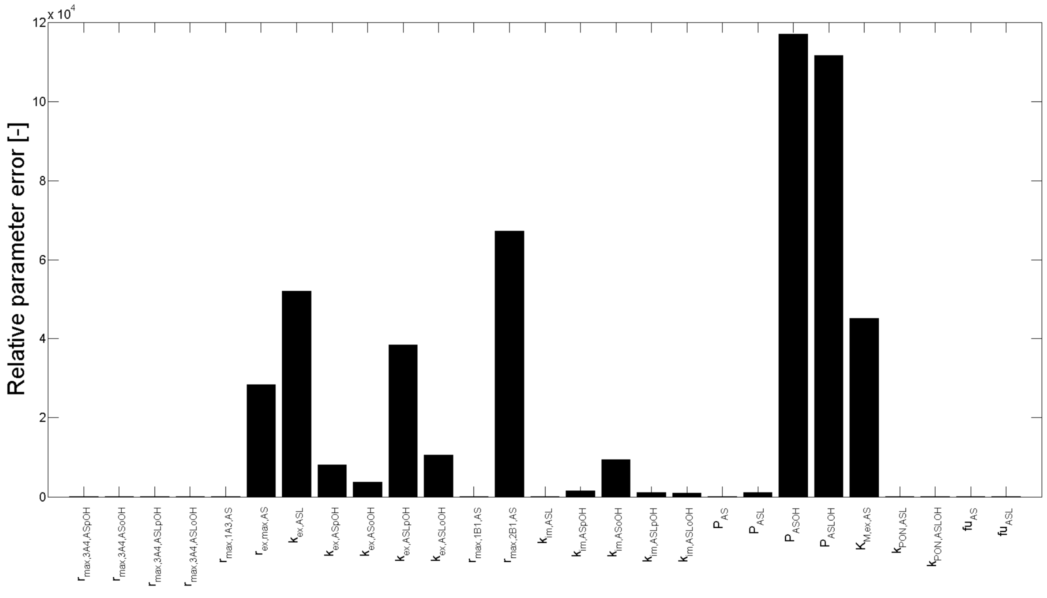

Figure C - Relative parameter errors of the full model of Atorvastatin metabolism

The relative parameter errors are determined from the FIM* analysis of the full model of Atorvastatin metabolism, comprising parameters which were estimated in the optimization procedure. High values indicate non-identifiable parameters.

Model reduction

The aforementioned problem poses the question whether identifiability can be improved by a rigorous model reduction. To achieve this, parameters showing very low normalized sensitivities, as described above, and corresponding transport steps, the export and the OATP2B1 mediated import of AS, were omitted. Next, in an iterative model reduction procedure, parameters with high relative errors and strong correlations in the covariance matrix were eliminated from the model. A new optimization step was performed on this reduced model and the new relative parameter errors and correlation matrix were estimated based on the **FIM*** analysis. In

this procedure, the permeability parameters P_{ASLOH} and P_{ASOH}, the active export parameter of ASL, $k_{ex,ASL}$, and the active import parameter of ASoOH, $k_{im,ASoOH}$, were deleted in the model. Further model reduction variants, for example the deletion of the import of ASL, led to a severe impairment in the objective function value and no satisfying fit in the re-optimization anymore, thus limiting further model reduction.

In summary, the reduced model was derived from the full model by eliminating parameters, $r_{max,ex,AS}$, $r_{max,2B1,AS}$, $K_{M,ex,AS,}$ P_{ASLOH}, P_{ASOH}, $k_{ex,ASL}$ and $k_{im,ASoOH}$, and corresponding transport steps, based on analysis of parameter sensitivity, correlations and errors.

The reduced model of atorvastatin biotransformation in primary human hepatocytes revealed 15 identifiable parameters of 20 total parameters in the top-down-classification of the **FIM***, which shows a reciprocal condition number of $2.84 \cdot 10^{-5}$. Thus, the condition number of the **FIM*** has been effectively improved by a factor greater than 10^{10}, indicating that the **FIM*** of the reduced model is much better scaled than the **FIM*** of the full model. Most important for the dynamic analysis, the maximal rate coefficients of the CYP3A4 catalyzed hydroxylation of AS and ASL to their respective metabolites and of the UGT1A3 catalyzed lactonization of AS could be considered to be identifiable in the top-down-classification.

The correlation matrix displays, that there are still high correlations in the model (**Figure D**), where 27 correlation coefficients are higher than 0.7, and most of them belong to metabolic reactions. This is most probably caused by the parallel pathway of CYP3A4-mediated hydroxylation of AS and ASL, UGT1A3 mediated lactonization of AS and hydrolysis of the lactone metabolites.

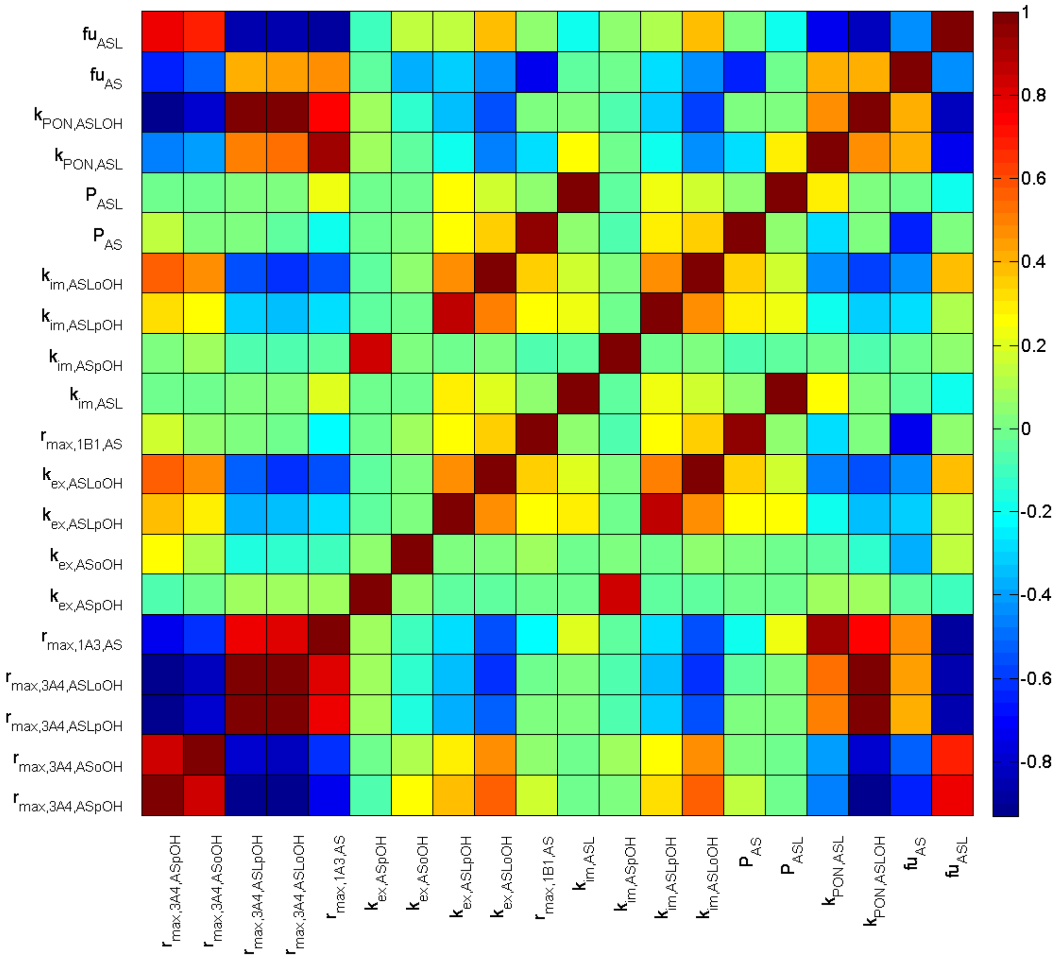

Figure D - Correlation matrix of the of the reduced model of Atorvastatin metabolism

The correlation matrix is displayed as pseudo-color plot of the parameters of the reduced model against themselves and comprises the single correlation coefficients between two parameters, respectively. Dark blue and dark red colors display parameters with high negative and positive linear dependencies, respectively.

In contrast to the full model, the relative parameter errors could be improved also by the model reduction procedure. The maximal relative parameter error equals 35% in the case of the parameter $k_{im,ASL}$, the rate coefficient of ASL import, and the minimal one equals 2 % in the case of $k_{ex,ASoOH}$, the rate coefficient of the ASpOH export (**Figure E**). In summary, the model reduction steps so far led to a drastic improvement in the local identifiability of the remaining optimized parameters, and thus strengthen the model reliability for predictions. The verified parameters after

model reduction procedure are summarized in **Table B** with nominal value and relative parameter errors from the **FIM*** analysis.

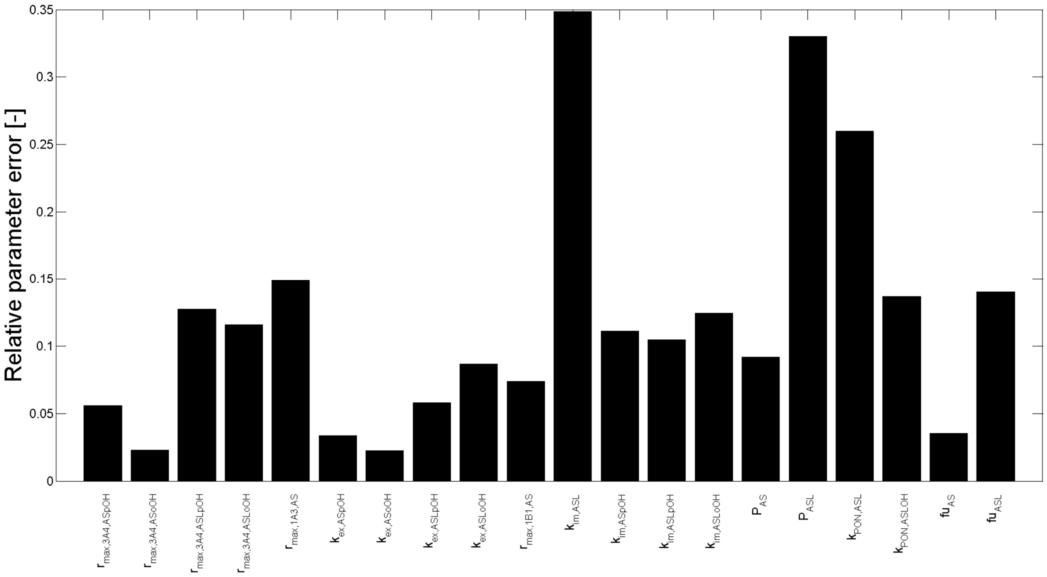

Figure E - Relative parameter errors of the reduced model of atorvastatin metabolism

The relative parameter errors are determined from the FIM analysis of the reduced model of atorvastatin metabolism after model reduction procedure. The lower the relative parameter error is, the more precisely the corresponding parameter could be estimated.

Table B - Verified parameters of the reduced model

Parameter	Value	rel. Error [%]	Units
$r_{max,3A4,ASpOH}$	1108.	5.5	pmol·min^{-1}·ml^{-1}
$r_{max,3A4,ASoOH}$	3345.	2.3	pmol·min^{-1}·ml^{-1}
$r_{max,3A4,ASLpOH}$	1228	12.7	pmol·min^{-1}·ml^{-1}
$r_{max,3A4,ASLoOH}$	2755	11.6	pmol·min^{-1}·ml^{-1}
$r_{max,1A3,AS}$	957	14.8	pmol·min^{-1}·ml^{-1}
$k_{ex,ASpOH,}$	0.81	3.3	µL·min^{-1}
$k_{ex,ASoOH}$	1.63	2.2	µL·min^{-1}
$k_{ex,ASLpOH}$	0.85	5.8	µL·min^{-1}
$k_{ex,ASLoOH}$	2.53	8.6	µL·min^{-1}
$r_{max,1B1,AS}$	461	7.4	pmol·min^{-1}
$k_{im.ASL}$	109	34.8	µL·min^{-1}
$k_{im,ASpOH}$	4.1	11.1	µL·min^{-1}
$K_{im,ASLpOH}$	21.4	10.4	µL·min^{-1}
$k_{im,ASLoOH}$	12.3	12.4	µL·min^{-1}
P_{AS}	2.4	9.1	µL·min^{-1}
P_{ASL}	9.7	33.0	µL·min^{-1}
$k_{PON,ASL}$	308	25.9	·10^{-3} min^{-1}
$k_{PON,ASLOH}$	280	13.6	·10^{-3} min^{-1}
fu_{AS}	0.22	3.5	-
fu_{ASL}	0.22	14.0	-

Parameters are listed with nominal values from optimization and relative parameter errors from **FIM** based identifiability analysis of the reduced model, in the corresponding units shown.

Literature

1. Baltes M, Schneider R, Sturm C, Reuss M: **Optimal Experimental-Design for Parameter-Estimation in Unstructured Growth-Models.** *Biotechnology Progress* 1994, **10:**480-488.
2. Faller D, Klingmuller U, Timmer J: **Simulation methods for optimal experimental design in systems biology.** *Simulation-Transactions of the Society for Modeling and Simulation International* 2003, **79:**717-725.
3. Joshi M, Seidel-Morgenstern A, Kremling A: **Exploiting the bootstrap method for quantifying parameter confidence intervals in dynamical systems.** *Metabolic Engineering* 2006, **8:**447-455.
4. Ljung L: *Sytem Identification: Theory for the User.* 2nd edn. New Jersey: Prentice Hall; 1999.
5. Munack A: **Optimization of Sampling.** In *Biotechnology - A Multi-Volume Comprehensive Treatise. Volume* 4. 2nd edition. Edited by Rehm HJ, Reed G, Schügerl K. Weinheim, New York: VCH; 1991: 251-264
6. Rodriguez-Fernandez M, Mendes P, Banga JR: **A hybrid approach for efficient and robust parameter estimation in biochemical pathways.** *Biosystems* 2006, **83:**248-265.

7. Zak DE, Gonye GE, Schwaber JS, Doyle FJ: **Importance of input perturbations and stochastic gene expression in the reverse engineering of genetic regulatory networks: Insights from an identifiability analysis of an in silico network.** *Genome Research* 2003, **13:**2396-2405.
8. Schneider R, Posten C, Munack A: **Application of linear balance equations in an online observation system for fermentation processes.** In *5th International Conference on Computer Applications in Fermentation Technology; Keystone, Colorado*. Edited by Karim MN, Stephanopoulos G. Pergamon Press; 1992: 319-322.
9. Rojas CR, Welsh JS, Goodwin GC, Feuer A: **Robust optimal experiment design for system identification.** *Automatica* 2007, **43:**993-1008.
10. Jacobsen W, Kuhn B, Soldner A, Kirchner G, Sewing KF, Kollman PA, Benet LZ, Christians U: **Lactonization is the critical first step in the disposition of the 3-hydroxy-3-methylglutaryl-CoA reductase inhibitor atorvastatin.** *Drug Metab Dispos* 2000, **28:**1369-1378.
11. Lau YY, Huang Y, Frassetto L, Benet LZ: **effect of OATP1B transporter inhibition on the pharmacokinetics of atorvastatin in healthy volunteers.** *Clin Pharmacol Ther* 2007, **81:**194-204.
12. Grube M, Kock K, Oswald S, Draber K, Meissner K, Eckel L, Bohm M, Felix SB, Vogelgesang S, Jedlitschky G, et al: **Organic anion transporting polypeptide 2B1 is a high-affinity transporter for atorvastatin and is expressed in the human heart.** *Clinical Pharmacology & Therapeutics* 2006, **80:**607-620.
13. Goosen TC, Bauman JN, Davis JA, Yu C, Hurst SI, Williams JA, Loi CM: **Atorvastatin glucuronidation is minimally and nonselectively inhibited by the fibrates gemfibrozil, fenofibrate, and fenofibric acid.** *Drug Metab Dispos* 2007, **35:**1315-1324.
14. Kearney AS, Crawford LF, Mehta SC, Radebaugh GW: **The interconversion kinetics, equilibrium, and solubilities of the lactone and hydroxyacid forms of the HMG-CoA reductase inhibitor, CI-981.** *Pharm Res* 1993, **10:**1461-1465.
15. Baker M, Parton T: **Kinetic determinants of hepatic clearance: plasma protein binding and hepatic uptake.** *Xenobiotica* 2007, **37:**1110-1134.
