# Supplementary material for: A systems biology approach to dynamic modeling and inter-subject variability of statin pharmacokinetics in human hepatocytes
Source: BMC Syst Biol. 2011 May 6;5:66. doi: 10.1186/1752-0509-5-66 (PMC3117731; doi:10.1186/1752-0509-5-66)
Supplement: Additional file 5 — Atorvastatin metabolite concentrations from the time-series experiment on primary human hepatocytes of individual 2. Extracellular concentrations (upper part) and intracellular concentrations (lower part) of atorvastatin acid and lactone (AS and ASL) and corresponding para- and ortho-hydroxy-metabolites (acids: ASpOH and ASoOH; lactones: ASLpOH and ASLoOH) at the defined time-points with mean and standard deviation (n = 3) from triplicate measurements per LC-MS/MS (n.d.: not determinable) (supplemented as .pdf-file). [file 1752-0509-5-66-S5.PDF]

Supplemental table S2: Atorvastatin metabolite concentrations in the time-series experiment on primary human hepatocytes of Individual 2

	AS		ASL		ASpOH		ASoOH		ASLpOH		ASLoOH	
time [min]	Extracellular Concentrations [pmol ml^{-1}]											
0	8803.3	±785.4	56.1	±5.1	n.d.		n.d.		n.d.		n.d.	
30	6709.7	±536.9	20.0	±2.8	2.1	±0.4	10.5	±1.8	n.d.		n.d.	
60	6416.5	±297.9	26.2	±1.5	10.0	±0.9	47.7	±1.5	1.3	±0.4	n.d.	
120	6571.1	±133.0	30.7	±2.2	77.7	±8.9	272.5	±10.8	3.4	±0.7	4.8	±0.2
240	5284.9	±288.6	40.4	±3.2	341.9	±29.8	875.2	±23.6	6.4	±1.0	9.4	±0.4
360	4676.7	±175.7	35.8	±2.0	561.9	±25.0	1348.3	±94.3	6.6	±1.0	9.7	±0.4
480	4271.2	±968.2	34.1	±1.9	754.1	±36.0	1666.4	±75.0	7.6	±1.1	10.4	±0.3
720	2626.6	±65.5	29.9	±2.1	1131.7	±82.2	2082.3	±93.1	10.2	±0.9	19.2	±0.9
time [min]	Intracellular Concentrations [pmol ml^{-1}]											
30	31162.9	±2469.3	350.3	±29.7	837.1	±19.4	592.4	±13.0	79.7	±4.8	n.d.	
60	29251.8	±2815.3	398.6	±29.8	1350.6	±16.6	857.0	±80.2	86.0	±2.8	n.d.	
120	30829.5	±98.1	459.0	±39.0	2634.3	±72.7	1784.9	±190.6	135.7	±5.1	32.4	±8.8
240	27117.8	±1142.2	518.2	±28.4	3165.4	±282.0	2077.5	±272.3	157.1	±10.0	51.5	±6.4
360	22432.9	±2525.3	422.5	±68.6	4342.4	±599.7	2489.7	±142.0	164.7	±21.8	63.8	±21.8
480	19098.5	±1589.6	359.7	±40.6	4045.3	±522.4	2704.5	±210.9	133.1	±9.8	45.8	±16.7
720	13392.5	±347.0	313.1	±22.5	5554.6	±463.9	3716.2	±459.9	148.1	±14.6	74.5	±12.2

Extracellular concentrations (upper part) and intracellular concentrations (lower part) of Atorvastatin metabolites, Atorvastatin acid and lactone (AS and ASL) and corresponding para- and ortho-hydroxy-metabolites (acids: ASpOH and ASoOH; lactones: ASLpOH and ASLoOH) at the defined time-points with mean and standard deviation (n=3) from measurements per LC-MS/MS (n.d.: not determinable; n.o.: not observed).
