# Supplementary material for: A systems biology approach to dynamic modeling and inter-subject variability of statin pharmacokinetics in human hepatocytes
Source: BMC Syst Biol. 2011 May 6;5:66. doi: 10.1186/1752-0509-5-66 (PMC3117731; doi:10.1186/1752-0509-5-66)
Supplement: Additional file 6 — Atorvastatin metabolite concentrations from the time-series experiment on primary human hepatocytes of individual 3. Extracellular concentrations (upper part) and intracellular concentrations (lower part) of atorvastatin acid and lactone (AS and ASL) and corresponding para- and ortho-hydroxy-metabolites (acids: ASpOH and ASoOH; lactones: ASLpOH and ASLoOH) at the defined time-points with mean and standard deviation (n = 3) from triplicate measurements per LC-MS/MS (n.d.: not determinable) (supplemented as .pdf-file). [file 1752-0509-5-66-S6.PDF]

**Supplemental table S3: Atorvastatin metabolite concentrations in the time-series experiment on primary human hepatocytes of Individual 3**

| | AS | | ASL | | ASpOH | | ASoOH | | ASLpOH | | ASLoOH | |
|---|---|---|---|---|---|---|---|---|---|---|---|---|
| time [min] | Extracellular Concentrations [pmol ml$^{-1}$] | | | | | | | | | | | |
| 0 | 9041.0 | ±195.5 | 27.8 | ±2.0 | n.d. | | n.d. | | n.d. | | n.d. | |
| 30 | 8344.6 | ±2257.8 | 27.0 | ±3.4 | n.d. | | n.d. | | n.d. | | n.d. | |
| 60 | 8580.2 | ±1810.9 | 32.1 | ±6.7 | 10.8 | ±0.2 | 28.7 | ±0.9 | n.d. | | n.d. | |
| 120 | 7439.3 | ±1331.9 | 51.6 | ±16.0 | 36.9 | ±2.9 | 90.4 | ±13.3 | 5.2 | ±0.1 | 6.0 | ±0.3 |
| 240 | 7035.6 | ±949.8 | 46.3 | ±12.7 | 224.7 | ±18.2 | 475.3 | ±33.9 | 7.0 | ±0.2 | 7.6 | ±0.6 |
| 480 | 4884.2 | ±652.5 | 60.6 | ±1.2 | 780.7 | ±26.4 | 1412.3 | ±25.4 | 10.1 | ±0.3 | 10.3 | ±0.8 |
| 720 | 4301.0 | ±153.6 | 38.7 | ±5.0 | 1087.5 | ±63.0 | 1747.6 | ±40.2 | 11.0 | ±1.3 | 12.1 | ±0.8 |
| 960 | 2530.3 | ±153.2 | 31.4 | ±10.2 | 1485.6 | ±69.7 | 2179.2 | ±76.0 | 14.5 | ±2.9 | 16.5 | ±3.9 |
| 1200 | 1968.0 | ±262.9 | 23.8 | ±2.7 | 1815.7 | ±46.9 | 2344.2 | ±87.5 | 18.5 | ±0.7 | 20.9 | ±0.9 |
| time [min] | Intracellular Concentrations [pmol ml$^{-1}$] | | | | | | | | | | | |
| 30 | 29613.3 | ±1767.5 | 389.8 | ±5.6 | 663.0 | ±19.3 | 433.5 | ±70.6 | 203.3 | ±3.0 | n.d. | |
| 60 | 38633.4 | ±5714.4 | 437.2 | ±55.1 | 3316.3 | ±536.8 | 1492.3 | ±169.8 | 222.6 | ±6.7 | n.d. | |
| 120 | 33630.5 | ±7944.7 | 483.7 | ±97.1 | 5683.9 | ±1294.6 | 2227.9 | ±640.3 | 243.9 | ±24.8 | 65.9 | ±20.5 |
| 240 | 31980.2 | ±1907.7 | 611.9 | ±58.6 | 7413.0 | ±462.1 | 2935.0 | ±462.1 | 297.5 | ±11.4 | 119.9 | ±50.9 |
| 480 | 27551.1 | ±3830.0 | 562.1 | ±101.0 | 10260.4 | ±2006.2 | 3724.9 | ±630.7 | 318.7 | ±34.5 | 147.2 | ±7.0 |
| 720 | 17601.2 | ±591.4 | 393.0 | ±57.1 | 8615.2 | ±590.5 | 2797.6 | ±576.7 | 275.4 | ±23.5 | 112.1 | ±48.1 |
| 960 | 13855.9 | ±1555.3 | 311.3 | ±17.0 | 12896.4 | ±875.7 | 3946.7 | ±466.6 | 323.5 | ±12.3 | 130.9 | ±67.3 |
| 1200 | 9622.1 | ±403.5 | 280.9 | ±23.4 | 15129.2 | ±1102.4 | 3628.3 | ±166.4 | 359.8 | ±23.6 | 152.8 | ±30.8 |

Extracellular concentrations (upper part) and intracellular concentrations (lower part) of Atorvastatin metabolites, Atorvastatin acid and lactone (AS and ASL) and corresponding para- and ortho-hydroxy-metabolites (acids: ASpOH and ASoOH; lactones: ASLpOH and ASLoOH) at the defined time-points with mean and standard deviation (n=3) from measurements per LC-MS/MS (n.d.: not determinable; n.o.: not observed).
